# How Vibrational Notations Can Spoil Infrared Spectroscopy: A Case Study on Isolated Methanol

**DOI:** 10.1021/acsphyschemau.4c00053

**Published:** 2024-10-04

**Authors:** Dennis F. Dinu, Kemal Oenen, Jonas Schlagin, Maren Podewitz, Hinrich Grothe, Thomas Loerting, Klaus R. Liedl

**Affiliations:** † Institute of Materials Chemistry, TU Wien, Getreidemarkt 9/165, Vienna 1060, Austria; ‡ Department of General, Inorganic and Theoretical Chemistry, University of Innsbruck, Innrain 80, Innsbruck 6020, Austria; § Department of Physical Chemistry, University of Innsbruck, Innrain 52, Innsbruck 6020, Austria

**Keywords:** infrared, spectroscopy, vibrational configuration interaction, matrix isolation, vibrational resonances, isotopic effect, nomenclature

## Abstract

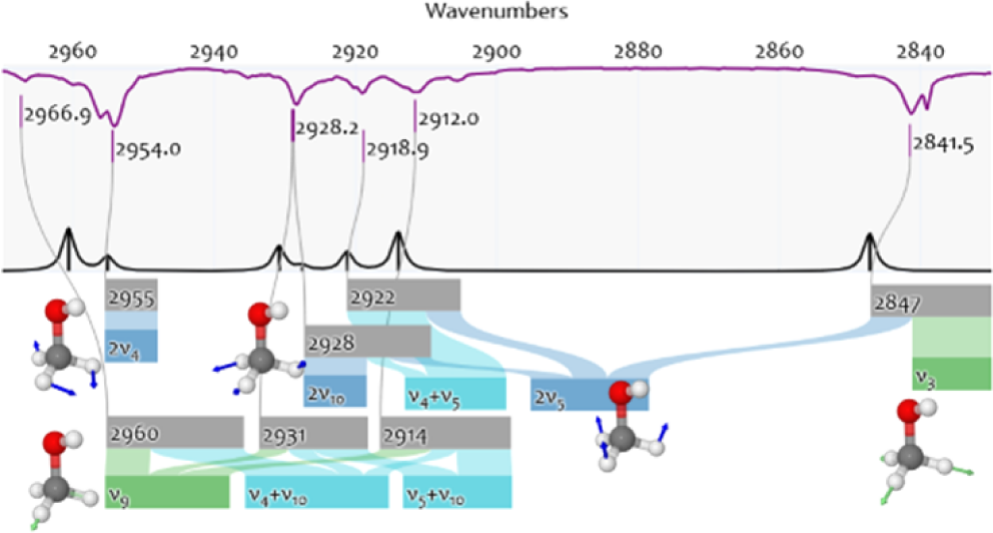

Unraveling methanol’s infrared spectrum has challenged spectroscopists for a century, with numerous loose ends still to be explored. We engage in this exploration based on experiments of isolating single methanol molecules in solid argon and neon matrices. We report infrared spectra of methanol in its natural isotopic composition and with partial and full deuteration. These experiments are accompanied by calculating wavenumbers involving anharmonicity and mode-coupling based on the vibrational configuration interaction approach. This allows for an unambiguous assignment of all fundamentals and resonances in the mid-infrared spectrum. An increasing degree of deuteration lifts resonances and aids in assigning bands uniquely. It also becomes evident that different notations typically used in chemistry or physics to describe molecular vibration from spectroscopy fail to describe the spectra appropriately. We highlight the shortcomings and suggest a more elaborate analysis using Sankey diagrams to unambiguously identify spectral features. Consequently, we demystify debated resonances occurring from various stretches and deformations of the methyl group.

## 1 Introduction

Methanol is omnipresent. It has always been an important driver for the chemical industry.^1^ Today, it is considered in the transition to sustainable energies,^2^ as it can be used as a combustion fuel^3^ and in direct methanol fuel cells.^4^ Methanol is a natural product in plant life.^5,6^ Its industrial and plant-based emissions lead to certain atmospheric methanol concentrations,^7,8^ where it can quench new particle formation.^9,10^ It takes part in human physiology,^11^ and as it can be poisonous,^12^ its detection in breathing gas is relevant.^13^ Furthermore, methanol is one of the few organic molecules identified in space, e.g., in interstellar clouds^14^ and on protoplanetary disks.^15^ Methanol is considered an important building block for forming larger molecules in space.^16^

For its detection in space, spectroscopy played a significant role for decades.^17^ This task requires reference spectra collected in the lab, understanding the bands’ origin, and identifying the molecular reason for their existence, i.e., the assignment of bands.^17^ This, in turn, necessitates theoretical models and a profound understanding of the approximations used in the theory.^18^ However, these approximations do not account for effects known in the experiment, such as band broadening or matrix splitting. This leads to *quantitative limitations*, i.e., the theory shows significant deviation from the experiment, and *qualitative limitations*, i.e., the theory cannot describe all experimental observations.

### 1.1 Concepts of Vibrational Spectroscopy

**Figure 1:**
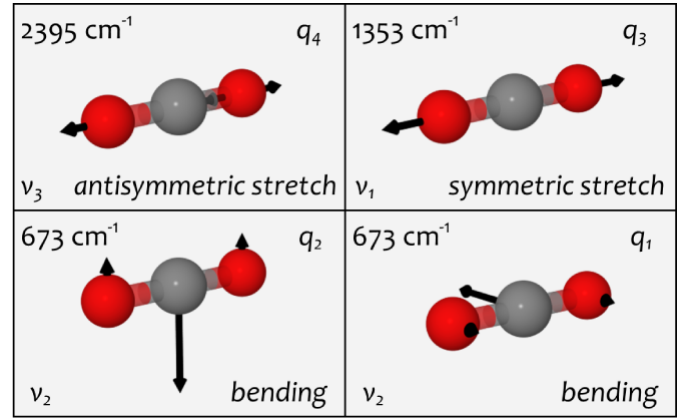
Normal modes of carbon dioxide as calculated in the harmonic approximation. Each panel shows a normal mode (*q*_i_, upper right), which is the basis of conventional vibrational notations using labels (ν_*i*_, lower left) or “trivial names” (lower right) to describe the molecular vibration, e.g., as ν_3_ or “antisymmetric stretch”. Each panel shows the corresponding calculated harmonic wavenumber (upper left) at CCSD(T)-F12/aug-cc-pVTZ-F12 level of theory.^22^

Many aspects of interpreting infrared (IR) and Raman spectra derive from the *harmonic approximation*. It dissects the external motion (rotation, translation) from the internal motion (vibration)^19^ and, subsequently, the vibrations into uncoupled harmonic oscillators.^20^ N atomic molecules comprise *3N-6* uncoupled vibrations, so-called *normal modes q*_*i*_. If the molecule is linear, it has *3N-5* normal modes. For illustrative purposes, Figure 1 shows the  normal modes of the linear carbon dioxide (CO_2_) together with the harmonic wavenumbers and a simplified vibrational notation.

The quantitative limitation of the harmonic approximation is its overestimation of vibrational wavenumbers.^21^ For example, in CO_2_, even when computed at a reasonably high level of theory^22^ the harmonic wavenumber of mode *q*_4_ overestimates the experimental value by about 45 cm^–1^. Figure 1 lists the harmonic *fundamentals*. Multiplying a fundamental with integers yields its *overtones*. Fundamentals and overtones add up to *combination bands*. With this procedure, one can predict the wavenumbers of overtones and combination bands as a linear combination of the wavenumbers of the fundamentals from the harmonic approximation. However, as the harmonic approximation overestimates the fundamental wavenumbers, the predicted wavenumbers for overtones and combination bands significantly deviate from the experiment. Furthermore, the harmonic approximation cannot predict their intensity.

The qualitative limitation of the harmonic approximation is reflected in conventional vibrational notations, some suggested almost a century ago.^23−25^ Such notations assume *3N-6* (or *3N-5*) vibrations, which sometimes cannot be uniquely assigned to experimentally observed bands. For example, the symmetric stretching vibration of CO_2_, or mode *q*_3_, should be observable in Raman spectroscopy. However, Raman experiments show two bands instead of one band,^26^ and both cannot be assigned to the symmetric stretching vibration of CO_2_.

To explain the Raman spectrum of CO_2_, Fermi assumed the quasi-degeneracy of the symmetric stretch vibration with the first overtone of the bending vibration^27^ and computed two wavenumbers in agreement with the experiment. Similarly, matrix-isolation IR spectroscopy of CO_2_ shows two bands in a region where the harmonic approximation does not directly predict any vibration.^22^ Only the assumption of a quasi-degeneracy of two combination bands, labeled as ν_3_+2ν_2_ and ν_3_+ν_1_, yields two wavenumbers in agreement with the experiment.^28^

**Figure 2:**
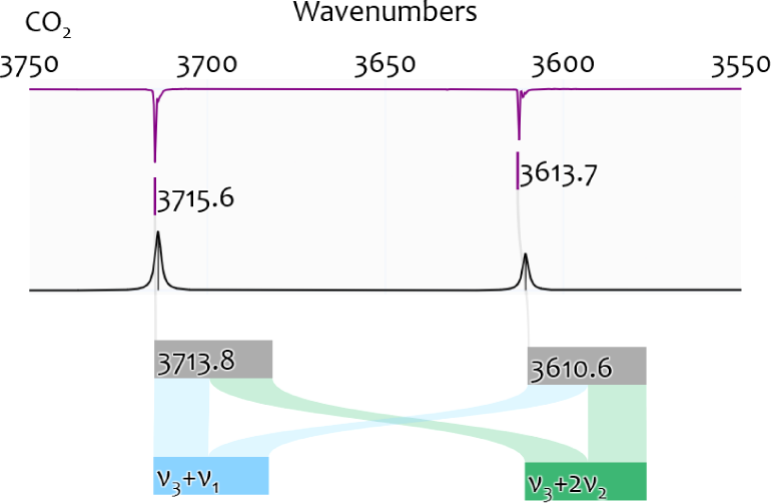
Matrix-isolation IR spectrum of CO_2_ in neon. The upper part shows the experimental spectrum on the top (purple) and the calculated spectrum on the bottom (black). The lower part shows the assignment as a Sankey diagram, illustrating the contributions of different vibrations (colored boxes) to the calculated wavenumbers (gray boxes) and their link to the experimentally observed wavenumbers (purple). Further details are given in Table S1.

We illustrate this assignment in the matrix-isolation IR spectrum of CO_2_ using a Sankey diagram in Figure 2. Sankey diagrams usually visualize a dynamic flow.^29,30^ Here, we use them to visualize a static mapping from a “label to a wavenumber.″ The diagram in Figure 2 shows that both “labels have almost equal contributions” to two computed wavenumbers, which can be directly assigned to two experimentally observed bands. The Sankey diagram shows that the notation of these bands is ambiguous, as both labels must be mentioned for either wavenumber. Such assignments are usually denoted as *resonance*.

### 1.2 Anharmonicity and Mode-Coupling

The two mentioned resonances in the CO_2_ spectra demonstrate the limitations of the harmonic approximation. The quasi-degeneracies observed in the experiment can be accurately matched using *anharmonic approaches*, with Fermi’s calculations relying on perturbation theory^27^ and our calculations relying on variational approaches.^22^ For details on various anharmonic approaches, we may refer to reviews.^31−38^ The harmonic approximation has two central issues: A) Bonds cannot be dissociated as the molecule’s potential energy goes to infinity with increasing bond length. Anharmonic approaches correct this by flattening the potential energy toward the dissociation energy. This is denoted as *anharmonicity*. B) The potential energy is modeled separately for each normal mode. Anharmonic approaches correct this by modeling the potential energy simultaneously for multiple normal modes. This is denoted as *mode-coupling*.

Various approaches include anharmonicity and mode-coupling, based on complex Hamiltonian forms that can provide high accuracy.^32,36,39,40^ In the approach we use here, the potential energy is computed along the molecule’s normal modes, which serve as coordinates.^41,42^ This N-mode expansion of the potential energy surface (PES) includes both anharmonicity and mode-coupling. It is used for solving the Schrödinger equation in a vibrational self-consistent field and configuration interaction (VSCF/VCI) approach.^43,44^ Normal coordinates facilitate calculations by allowing for simple Hamiltonian forms while obtaining reasonable accuracy^42,45^ and retaining conventional vibrational notations. This preserves ease of interpretation from the harmonic approximation while increasing rigor in the theoretical model. However, it remains intricate to ultimately answer the question “Is ease of interpretation sacrificed as rigor increases?″ as formulated by Qu and Bowman.^46^

This is because the issue of interpretation is often connected to the term *resonance* mentioned above. It usually indicates the coincidence of frequencies in oscillating systems. In the context of molecular vibration, however, experimentally observed frequencies associated with a resonance do not coincide. Here, the resonance merely assumes a quasi-degeneracy of vibrations. Yet, subsequent “mixing” of these vibrations yields the experimentally observed frequencies.^27^ This renders vibrational resonances somewhat obscure in their interpretation. Additionally, resonances differ for each molecule, cf. Fermi resonance in CO_2_,^27^ Darling-Dennison resonance in H_2_O.^47^

Today, the terms Fermi resonance and Darling-Dennison resonance have broader applications. Fermi resonances are commonly used to indicate a quasi-degeneracy between a fundamental and a first overtone or between a fundamental and a binary combination band. Darling-Dennison resonances indicate more complex quasi-degeneracies, e.g., involving a combination band with other combination bands and first overtones.^48^ While the resonances in CO_2_ are relatively simple, they become more challenging to grasp for larger molecules, especially when multiple X-H bonds (X = O, C, N) are present. This brings us back to methanol, which combines a methyl group (C–H bonds) and a hydroxyl group (O–H bond), two common functional groups in many organic molecules. For methanol, anharmonic approaches demonstrated promising quantitative agreement with the experiment,^28,49^ especially with matrix-isolation infrared (MI-IR) spectroscopy.^28^ However, the qualitative agreement from such calculations is sparsely investigated, with few studies on resonances.^50,51^

Clarifying the vibrational spectrum of methanol, including its resonances, is an ongoing challenge. To tackle this challenge, we have recorded a comprehensive experimental data set of MI-IR spectra of CH_3_OH, CH_3_OD, and CD_3_OD in both Argon and Neon matrices at various dilutions. VSCF/VCI calculations on an N-mode PES expansion, including anharmonicity and mode-coupling, allow us to recapitulate the assignment. Consequently, we aim for an improved qualitative agreement with the experiment, including the assignment of fundamentals, combination bands, overtones, and resonances within conventional vibrational notations.

### 1.3 Historical Overview on the Infrared Spectroscopy of Methanol

**Figure 3:**
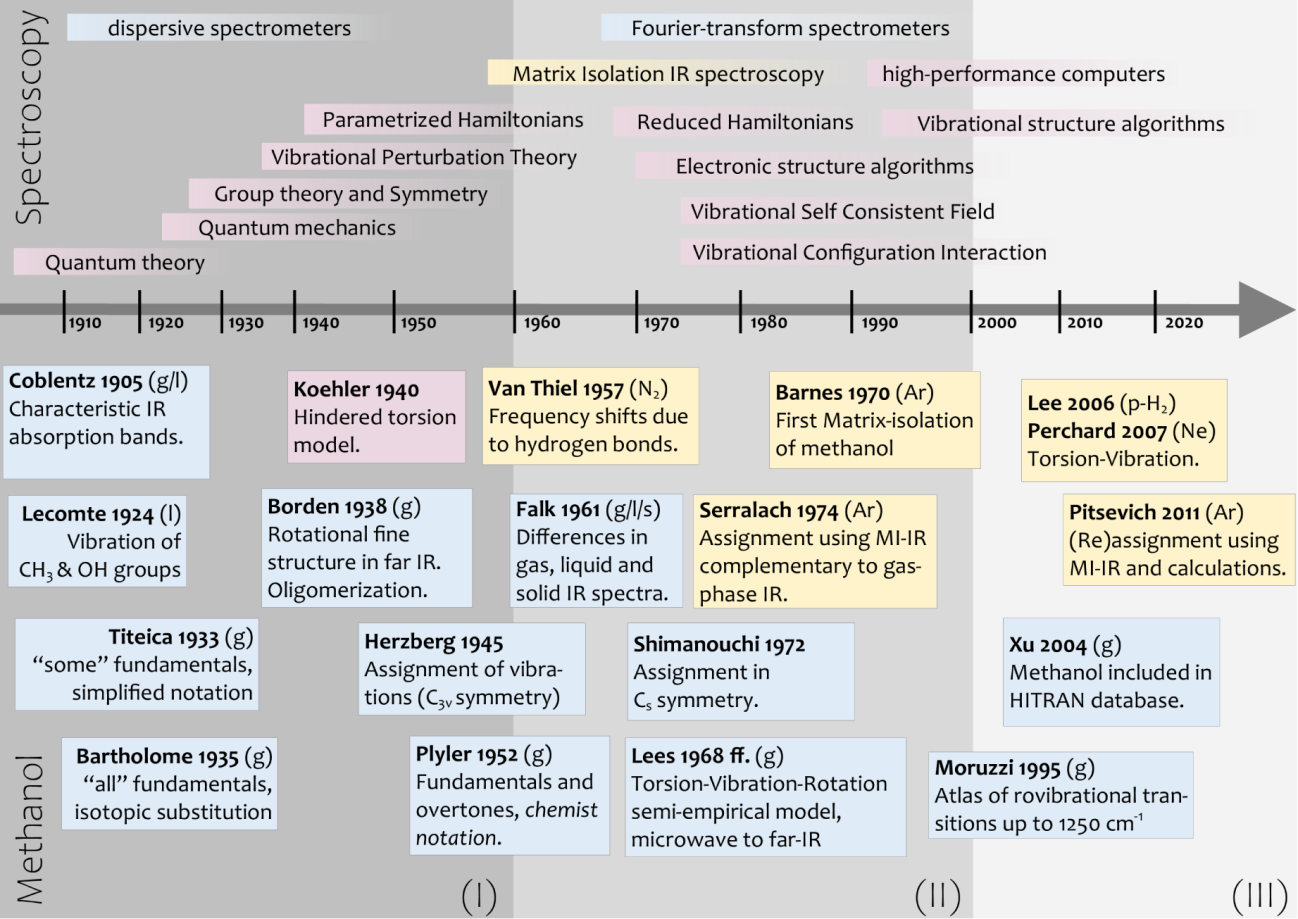
Historical overview on the IR spectroscopy of methanol. The highlighted studies on the lower part of the timeline focus on the advances in the assignment of the fundamental vibrations of methanol in gas/liquid/solid phase (g/l/s) or in matrix isolation (N_2_, Ar, etc.). Details on other aspects are elaborated on in the main text. Major developments in theoretical and experimental spectroscopy are given in the upper part of the timeline for better historical guidance. We divide the achievements into three time-frames: (I) Advent of quantum theory to describe low-resolution spectroscopic experiments. (II) Progress toward high-resolution laboratory spectroscopy. (III) Computations approaching “spectroscopic accuracy”.

Figure 3 illustrates the history of IR spectroscopy focusing on methanol, divided into three frames. The beginnings (I) were dominated by low-resolution spectroscopy and the advent of quantum mechanics. This was followed by improvements in spectroscopic experiments (II) and has led to highly accurate spectroscopic information (III) from theory and experiment.

**(I)** IR absorption of “methyl alcohol” as an organic liquid was first studied more than a century ago.^52−54^ In 1904, molecular vibration was merely a hypothesis for diatomic molecules.^55^ Hence, assigning certain spectral features in larger molecules was tentative. In the 1920s, some bands in the IR spectrum of methanol were vaguely attributed to either alcohol or methyl groups.^56,57^

During the 1910s, early quantum theory for atoms (cf. Bohr’s atomic theory^58^) introduced useful concepts for interpreting spectra of diatomics.^59−61^ This has led to explaining IR spectra as harmonics of rotational–vibrational transitions,^62^ including also anharmonic corrections.^63^ Considering the IR spectrum of methanol, the first theoretical discussion was given in 1929, albeit without sophisticated assignments.^64^

During the 1920s, early quantum theory^65^ evolved into quantum mechanics,^66,67^ which was subsequently adapted for molecules.^68,69^ In 1931, Dennison advised against relying on harmonic models for interpreting IR spectra of polyatomic molecules.^69^ Nevertheless, in 1933, such models enabled the first assignments in the IR spectrum of methanol,^70^ using a simplified notation for the experimentally observed wavenumbers.

At about the same time, quantum mechanics and molecular symmetry were linked via group theory.^71^ During the 1930s, group theory for molecular rotation and vibration^72,73^ paved the way for notations using symmetry labels and established the connection between spectroscopy and molecular structure. In 1938, Borden and Barker resolved rotational transitions of methanol in gas-phase^74,75^ and confirmed it behaving as an asymmetric top.^69,76^ In 1940, the aspect of hindered torsion was proposed, where the staggered conformation features a 3-fold degenerate energy minimum.^77^ Subsequent experiments relied on this model for categorizing the observed vibrational bands into 12 fundamentals of two irreducible representations.^78^

Herzberg captured many findings up to 1945 in his prominent encyclopedia.^79^ Considering methanol, it shows significant discrepancies to the catalog provided by Shimanouchi^80^ in 1972. While Herzberg’s methanol assignment relied on sparse experimental references and used the notation in analogy to acetonitrile CH_3_CN in the *C*_*3v*_ point group with 8 fundamentals, Shimanouchi provided a more consistent assignment for the 12 fundamentals of CH_3_OH in the correct *C*_*s*_ point group. These seminal works are somewhat outdated, as high-resolution spectroscopy arose in the second half of the 20th century.

**(II)** Around 1940, it was known that gas-phase IR spectra are complicated by rotational–vibrational interactions^74^ and that liquid-phase IR spectra exhibit broad bands due to the formation of oligomers, i.e., hydrogen-bonded methanol entities.^81^ Retrospectively, it is clear that two paths for retrieving further information from IR spectroscopy were necessary from this point onward: (a) Probing solid-, liquid-, and gas-phase to inspect their influence on the IR spectra. (b) Improving the resolution of spectrometers.

The wavenumber shifts due to hydrogen bonding were studied in liquids around the 1950s.^82−84^ By 1961, Falk and Whalley counted roughly 20 studies that dealt with methanol in various liquid- and gas-phase experiments.^85^ They also investigated solid methanol to clarify the difference between monomer and bulk. A significant advance was made in 1954 based on matrix isolation infrared (MI-IR) spectroscopy.^86^ Both rotation and oligomerization could be experimentally quenched at high dilution and low temperature. Van Thiel et al. investigated the first MI-IR spectra of methanol in N_2_ matrix in 1957, revealing wavenumber shifts due to oligomerization and successfully distinguishing OH stretch and CH_3_ stretch vibrations.^87^

In 1970, Barnes and Hallam provided the first complete assignment of vibrational transitions in the mid-IR region of MI-IR spectra (Ar matrix), considering monomers, dimers, and oligomers for most isotopologues.^88^ Simultaneously, Mallinson and McKean provided MI-IR spectra (Ar matrix) and derived a molecular force field to calculate vibrational wavenumbers.^89^ The early MI-IR studies culminated in the exhaustive study by Serrallach et al. from 1974, who adapted information from MI-IR spectroscopy to assign the corresponding bands in the gas-phase spectra.^90^ The assignment of methanol’s vibrational fundamental transitions presented therein can be considered the most rigorous until today.

Considering gas-phase spectroscopy, during the 1950s, progress in the microwave and far IR spectroscopy played a dominant role.^91−94^ Deriving the molecular structure of methanol from such experimental data was promoted,^95−97^ and the theoretical model for the hindered internal rotation, or torsion, was refined.^98,99^ Around 1970, Lees scrutinized the torsion-vibration–rotation interactions in methanol using millimeter/microwave spectroscopy^100−103^ and proposed an elaborate spectroscopic notation for these interactions.^104^ At about the same time, gas-phase spectroscopy in selected mid-IR regions arose,^105,106^ albeit with a mediocre resolution by today’s standards.

Likely, the suggestion of methanol as a laser-active medium in 1970^107^ encouraged a deeper understanding of rovibrational transitions, thus further promoting high-resolution spectroscopy. From the early 1980s onward, Moruzzi et al. extensively investigated and assigned the far- and mid-IR region using high-resolution Fourier transform spectrometers.^108−114^ By 1995, they provided an atlas for rovibrational transitions for the spectroscopic region up to 1258 cm^–1^.^115^

While gas-phase spectroscopy evolved toward high-resolution experiments to derive accurate models, including torsion, rotation, and vibration, matrix-isolation spectroscopy was particularly successful in investigating pure vibration. Since the 1980s, many MI-IR studies of methanol have been published, using different host materials such as Nitrogen (N_2_),^116−126^ Argon,^119−122,124,125,127−133^ Neon,^123,134−136^ and para-Hydrogen (p-H_2_).^137^ Many MI-IR studies focus on methanol clusters^116,118,119,124−126,130,131,135,136,138,139^ and clusters with water or other molecules.^117,120,121,128,129^ Alternatively, IR spectra of such clusters were studied via supersonic jet expansion^140,141^ and the doping of Helium nanodroplets.^142,143^

**(III)** In 2004, parts of the methanol spectrum were introduced to the HITRAN database,^144^ marking a milestone in the availability of high-resolution gas-phase spectra. For a relatively recent systematization of the enormous amount of spectral data and different notations considering methanol, we may refer to a study from 2017.^145^ The increasing availability of high-resolution spectra pushes contemporary research toward *spectroscopic accuracy*, e.g., by using tailor-made theoretical models for certain parts of the gas-phase spectrum.^146^ Spectroscopic accuracy is achieved “when the errors of calculations are significantly smaller than a typical distance between vibrational band centers that makes possible an unambiguous assignment of bands in observed spectra” (from Nikitin et al.^147^)

For MI-IR spectra, spectroscopic accuracy in the theoretical model is achieved when all vibrations can be distinguished.^22,28,148,149^ Calculations on methanol that were accomplished in the early 2000s using different approaches^49,50,150−152^ lowered the mean absolute deviations to about 4 cm^–1^.^28^ This accuracy can be expected to be sufficient for evaluating MI-IR spectra in the mid-IR region. Aside from that, newer developments using curvilinear coordinates have been evaluated for methanol.^153,154^ We may refer to a recent work on the variational vibrational states of methanol for more details on such developments.^155^ Torsion-vibration interactions prevail in MI-IR spectra, as reported in 2006 by Lee et al. for para-H_2_ matrices^137^ and subsequently by Perchard in Ne matrices.^134^ The latter also explained some resonances based on perturbation theory.^51,123^

Today, one of the challenges for theory is explaining molecular vibration in the complete mid-infrared spectrum.^31−38^ The present work tackles this challenge for methanol by combining experimental MI-IR spectroscopy with calculations for molecular vibration, including anharmonicity and mode-coupling, in a VSCF/VCI approach.^28^

## 2 Results and Discussion

### 2.1 Conventional Vibrational Notations

In gas-phase IR spectroscopy of methanol, rotation-torsion-vibration interactions evoke a complex spectrum.^100,104^ For such spectra, a notation for all observed bands includes the vibrational quantum number, a specific vibrational quantum number for the internal torsion, the torsional symmetry, and the two rotational quantum numbers of an asymmetric top.^104^ For matrix-isolation IR spectroscopy of methanol, rotation is quenched, which leads to a somewhat simpler notation. Still, torsion-vibration interactions occur^134,137^ and can be labeled analogously to gas-phase IR. We discuss these torsion-vibration interactions in the Supporting Information and focus on vibrational notations in the following.

**Figure 4:**
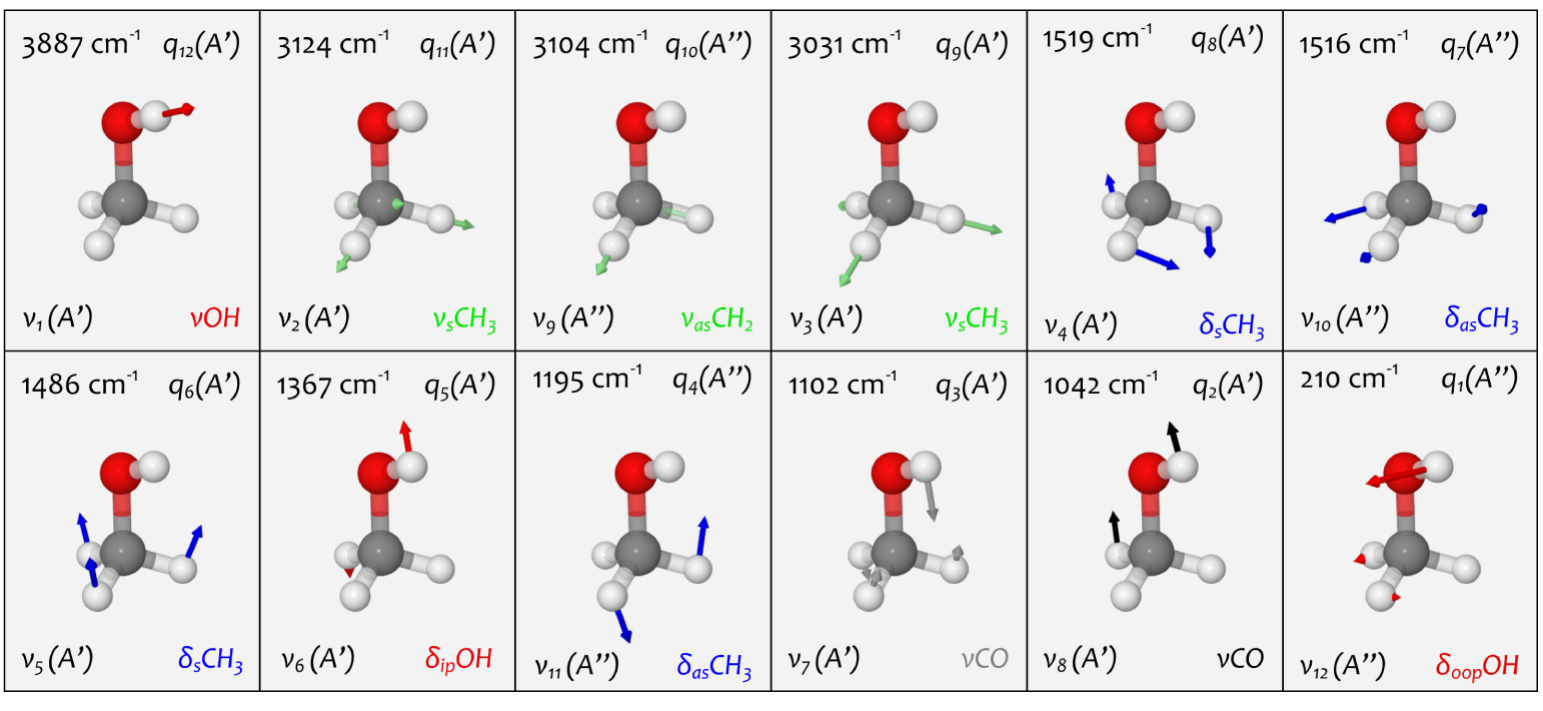
Normal modes *q*_*i*_ of methanol (CH_3_OH) in the *C*_*s*_ point group. Each panel depicts one normal mode together with its harmonic oscillator wavenumbers computed at CCSD(T)-F12/VTZ-F12 level of theory (upper left) and its labels from the computational notation (upper right), the spectroscopist notation (lower left) and the chemist notation (lower right) highlighted through a color code: OH vibrations (red), CH stretch vibrations (green) CH deformations (blue), CO stretch vibrations (gray, black).

Normal modes and harmonic wavenumbers are obtained from diagonalization of the matrix of second derivatives for the energy w.r.t. Cartesian atomic displacements (Hessian).^20^Figure 4 depicts the normal modes *q*_*i*_ of methanol with their harmonic wavenumbers and labels from *conventional vibrational notations*, which we denote as “conventional” because they are prevalent in the scientific community. The different notations have various benefits and drawbacks from a computational, spectroscopy, chemistry, or physics perspective so that they can be categorized by these contexts. A combination of notations is likely the most descriptive and accurate way of assigning the experimental spectrum. However, such a combined notation is cumbersome unless a clear strategy, as will be suggested here, is followed. Table 1 lists for each normal mode *q*_*i*_ of CH_3_OH the labels from the computational, spectroscopist, physicist, and chemist notation. Additionally, it shows the principal motion patterns from visually inspecting the normal modes and from normal mode decomposition.^156^ We discuss several aspects (A-F) considering the notations in the following.

**Table 1:** Conventional Vibrational Notations of Methanol’s Fundamental Vibrations Derived from the Normal Modes of CH_3_OH in the *C*_*s*_ Point Group

Vibrational notations					Principal motion patterns^a^	
Normal mode	Computational	Spectroscopist	Physicist	Chemist	Visual	Decomposition
				*νOH*	OH bond	100% rOH
				*ν_s_CH_3_*	CH_3_ bonds	78% rCH_σ_ + 22% rCH_ϵ_
				*ν_as_CH_2_*	CH_2_ bonds	100% rCH_ϵ_
				*ν_s_CH_3_*	CH_3_ bonds	78% rCH_ϵ_ + 21% rCH_σ_
				*δ_s_CH_3_* scissor	CH_3_ angles	67% αH_ϵ_ CH_ϵ_ + 30% αH_σ_ CH_ϵ_
				*δ_as_CH_2_* rocking	CH_2_ angle	92% αH_σ_ CH_ϵ_
				*δ_s_CH_3_* umbrella	CH_3_ angles	46% αOCH_ϵ_ + 36% αH_σ_ CH_ϵ_ + 16% αH_ϵ_ CH_ϵ_
					COH angle	68% αHOC + 16% αOCH_ϵ_ + 13% αH_ϵ_CH_ϵ_
				*δ_as_CH_3_* twisting	CH_3_ angles	94% αOCH_ϵ_
				scaffold	CO bond	72% rCO + 14% αHOC + 9% αOCH_ϵ_
					CO bond	41% rCO + 29% αHOC + 28% αOCH_ϵ_
				torsion	COH angle	96% τHOCH_σ_

a Principal motion patterns were derived by visually inspecting the normal mode vectors (cf. Figure 4) and normal mode decomposition using nomodeco(156) (cf. Figure S1). Only the highest contributions (>9%) are shown here. H_σ_ refers to the hydrogen in the mirror plane and H_ϵ_ to the two hydrogen atoms out of this plane.

(A) The normal modes *q*_*i*_ are sorted by their harmonic wavenumber, with the index *i* increasing from lowest to highest wavenumber, as obtained from the diagonalization of the Hessian. The order of the indices is usually inversed for better comparison with conventions in the experiments, where the spectrum is studied from highest to lowest wavenumber. In computational studies, the calculated harmonic wavenumber is often labeled as ω_*i*_. We may call this the **computational notation**. The subscript *i* inherits the numbers from the normal mode *q*_*i*_, including the inversed order of the indices. Methanol’s vibration with the highest wavenumber of 3887 cm^–1^ is labeled *ω*_1_. As the order of the indices is somewhat arbitrary, comparing different studies using a computational notation can be confusing. Furthermore, the symmetry information is often neglected in such notations. Hence, these computational notations are not recommended for communicating assignments in IR spectroscopy.

(B) Each normal mode *per se* involves motions of all atoms. However, some atomic displacements dominate the overall nuclear motion. We may call these displacements **principal motion patterns**. Each vibration can be associated with a “trivial name” based on the principal motion pattern. This results in the **chemist notation**, preferred in chemically motivated spectroscopy, where the notation should illustrate the moiety involved in the vibration and its type of motion. Such notation was early proposed by Mecke in the 1930s.^23−25,157,158^ For example, the methanol vibration at 3887 cm^–1^ is labeled , an abbreviation for “OH stretch vibration.″ The issue with these principal motion patterns is that they are often subjectively identified by ′looking at′ normal modes, either by plotting the normal mode vectors (as in Figures 1 and 4) or animating them through visualization software. Alternatively, certain molecule parts’ contribution to the normal mode can be quantified, e.g., by local mode theory^159^ or potential energy distribution (PED) analysis.^160−165^ PED schemes enable the decomposition of normal modes to contributions from *primitive internal coordinates*, such as bonds, angles, and dihedrals. For this purpose, we use the recently developed nomodeco tool from our group.^156^Table 1 lists the principal motion patterns for methanol. The principle motion is located at the OH bond in a visual inspection of the normal mode *q*_12_ (cf. Figure 4). This agrees with a contribution of 100% from the internal coordinate rOH. Visual inspection of the modes *q*_2_ and *q*_3_ shows they are delocalized along three internal coordinates (cf. Figure 4). We see large contributions from rCO for both *q*_2_ (41%) and *q*_3_ (72%). Usually, the *q*_2_ mode is labeled as . However, based on nomodeco, we could also label the *q*_3_ mode as .

(C) As the molecular symmetry is connected to the spectrum, we name a notation that includes the molecular point group symmetry as **spectroscopist notation**. Each normal mode is associated with an irreducible representation (*irrep*) of the molecular point group. In the spectroscopist notation, the normal modes *q*_*i*_ are binned by their *irrep*. These bins are sorted from highest to lowest *irrep*, as given by the respective character table of the point group. This ordering is reflected in the symbols used for the *irrep* in the Mulliken symbols:^166^*A* is symmetric regarding the highest symmetry axis, *B* follows as antisymmetric, etc. Other symbols (*A1*, *A2*, *A'*, *A″*, *A*_*g*_, *A*_*u*_, etc.) indicate symmetry/antisymmetry regarding the consecutive symmetry operations in the point group, as categorized in the character table. The normal modes within an *irrep* are sorted by their harmonic wavenumber in descending order. For methanol in the *C*_*s*_ point group, the irrep is either *A'* or *A″*, with *A'* being of higher symmetry than *A″*. Each vibration has a label , and the subscript *i* is counted starting with 1. In this notation, the methanol vibration at 3887 cm^–1^ has the label . The benefit of this notation is that the *irrep* directly provides symmetry information that can be translated into IR/Raman activity of the corresponding transition.

(D) The spectroscopic notation can be confusing regarding **isotopic exchange**. As shown in Figure 5, the vibrational wavenumber can considerably change upon isotopic exchange, especially when substituting hydrogen with deuterium. Thus, the order of the vibrations changes significantly. Consequently, the subscripts in the spectroscopic notation differ for different isotopologues. In contrast, the chemist notation does not rely on any numbering and can include the isotopologue in the notation. In CH_3_OH,  has a higher wavenumber than . In CH_3_OD,  has a lower wavenumber than . That means that when comparing the stretch vibrations  and  within the spectroscopist notation, the correct labels to compare are  for CH_3_OH with  for CH_3_OD. Sometimes, this subtlety is not obeyed, and the labels from the most abundant isotopologue are generally used for all isotopologues. Consequently, the spectroscopic notation can become misleading.

**Figure 5:**
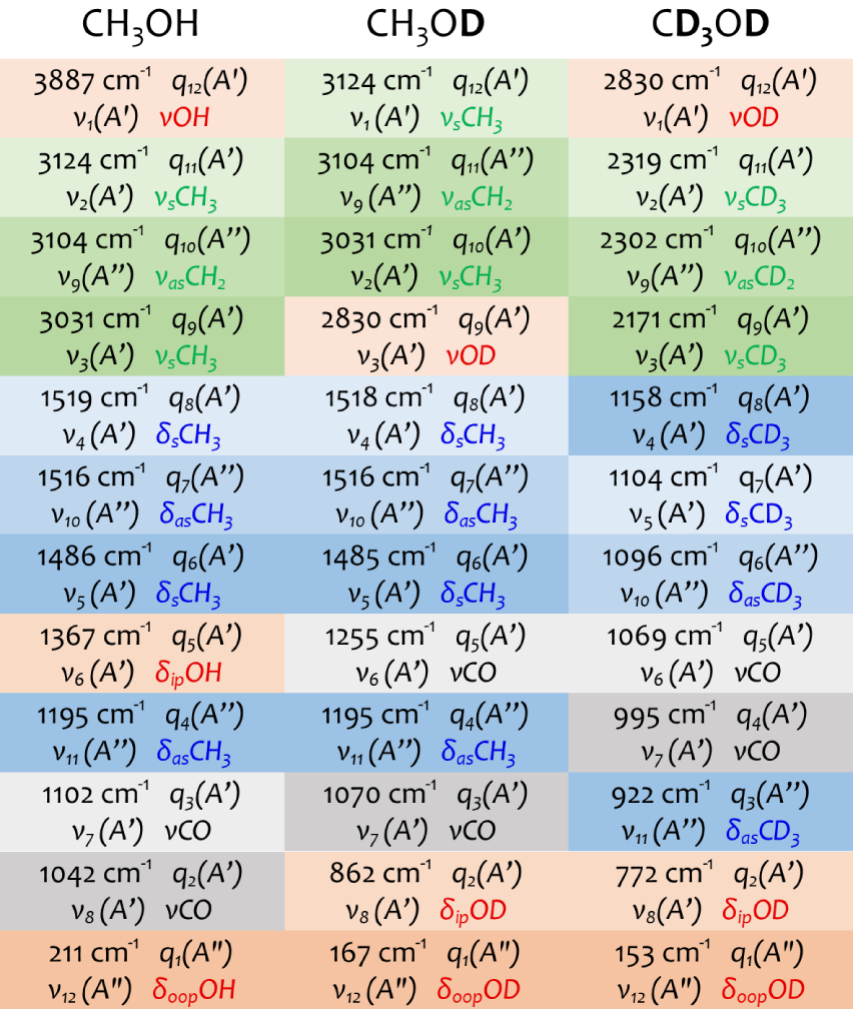
Influence of isotopic exchange on the vibrational notations of CH_3_OH, CH_3_OD, and CD_3_OD. Each panel shows the harmonic wavenumber (upper left) and its labels from the computational notation (upper right), the spectroscopic notation (lower left), and the chemist notation (lower right) using the color code as in Figure 4.

(E) As spectroscopy relates to quantum mechanics, notations may directly carry quantum mechanic information. These **physicist notations** are usual in evaluating high-resolution spectra, where tailor-made models must be set up, and the resulting quantum numbers and notations are molecule-specific (cf. Handbook of high-resolution spectroscopy^167^). However, certain approaches for semirigid polyatomic molecules can retain the above-mentioned notations. An example is the VSCF/VCI approach based on N-mode PES, as implemented, e.g., in the Molpro software package^168^ by Rauhut et al.^38,42,45,169−173^

In VSCF, the wave function is a product of one-mode functions, which depend on one vibrational degree of freedom described by one normal mode coordinate *q*_*i*_. Such products are called *configurations* and are written in the Dirac braket formalism. For example, the configuration  is a wave function where only the one-mode function of normal mode *q*_12_ is occupied. Each VSCF calculation yields one specific configuration with a corresponding VSCF state energy that uniquely maps to the conventional vibrational notations. For methanol, the  configuration is mapped to  or . While it is common to write out the configurations as vectors,^34^ e.g., the short-hand notation  would be referred to as , we remain with the short-hand notation here. In VCI, the wave function is a linear combination of configurations. If one configuration dominates a VCI wave function, mapping to conventional vibrational notations is unique and unproblematic. If the configuration , a single excitation in the one-mode function of normal mode *q*_9_, has a high contribution, the state is denoted as ω_4_ in the computational notation, as  in the spectroscopist notation, or as the ν_*s*_CH_3_ fundamental in the chemist notation. Similarly, if the configuration , a double excitation in the one-mode function of normal mode *q*_8_, has a high contribution, the state is denoted as , , or as the first overtone of δ_*s*_CH_3_.

(F) When unique mappings are impossible, we need to consider **resonances**. If two configurations have similarly high contributions to a state , e.g., 34% of  and 28% of , it is impossible to map one specific label from a conventional notation to . We say that  is in *resonance* with (at least) one other state  under the assumption that they are quasi-degenerate, i.e., similar in energy. If  is computed, state  can be found by analysis of all significant contributions of configurations to all states in a defined wavenumber range next to the wavenumber of . Then,  may have similarly high contributions from two configurations, e.g., 32% of  and 36% of . This translates into a **resonance** between ω_4_ and  in the computational notation, or  and  in the spectroscopist notation, or the ν_*s*_CH_3_ fundamental and the first overtone of δ_*s*_CH_3_ in the chemist notation. Two experimentally observed wavenumbers that quantitatively agree with this resonance cannot be uniquely assigned using either of the conventional vibrational notations. At this point, one can resort to analogies with the Fermi resonance in CO_2_^27^ or the Darling-Dennison resonance in H_2_O.^47^ Alternatively, the contributing configurations from VCI can be mentioned, e.g., by Sankey diagrams (cf. Figures 2 and 7).

### 2.2 Assignment of Vibrations in the Mid-IR Spectrum of Methanol

Table 2 lists the most prominent effects that can complicate a mid-IR spectrum of methanol. In the gas-phase IR spectrum of methanol, *rotation* induces partially overlapping rotational–vibrational lines^74^ and *torsion* causes further line splittings.^100^ Also, *resonances* must be considered. Matrix isolation evades some of these complications. *Rotation* can be avoided completely, while *torsion* splitting was observed in Ne and *p*-H_2_ matrices^134,137^ but not in Ar matrices. Matrix isolation favors aggregation of multiple methanol molecules (*oligomerization*),^174^ yet high dilutions minimize this. Consequently, in MI-IR spectroscopy, *resonances* and *matrix effects* remain at least.

**Table 2:** Differences Between IR Spectroscopy of Methanol in the Gas Phase and Matrix Isolation

	gas phase	matrix isolation^a^
rotation	yes	no
torsion	yes	yes (Ne)/no (Ar)
oligomers	no	no (high dilution)
matrix effects	no	yes
resonances	yes	yes

a All aspects depend on the specific host-guest system.^28^

**Figure 6:**
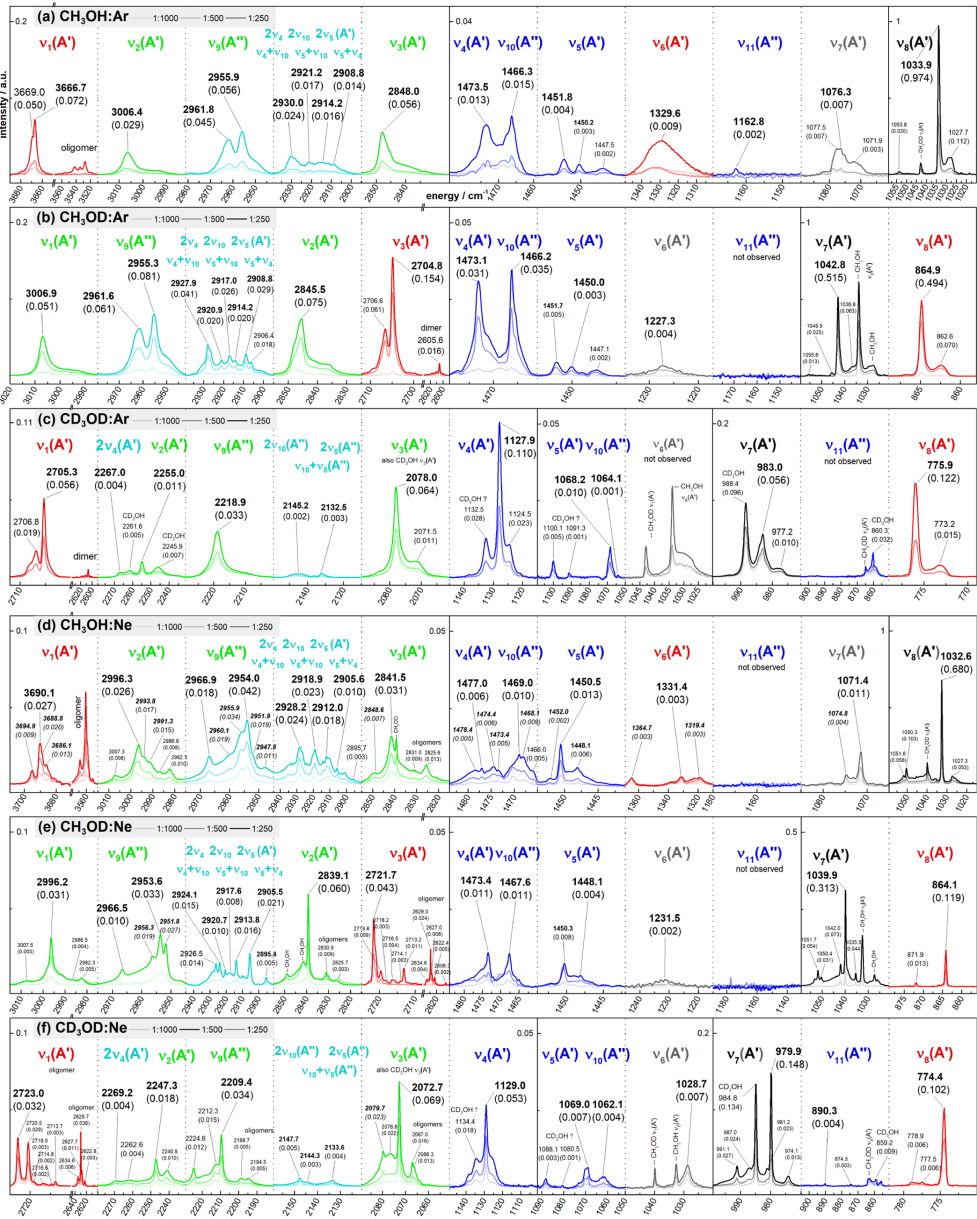
Mid-IR spectrum of CH_3_OH, CH_3_OD, and CD_3_OD isolated as matrices at 6 K using argon (a)–(c) and neon (d)–(f) in different dilutions of analyte:matrix (1:1000, 1:500, 1:250). The spectral regions are labeled by the spectroscopist notation and colored by the chemist notation (cf. Figure 4). Selected wavenumbers (in cm^–1^) are shown together with their intensities (in normalized arbitrary units) given in brackets. Assigned wavenumbers are in bold letters.

Compared to calculations, one must decompose anharmonicity and mode-coupling from the matrix effects.^22^ Matrix effects are related to the local host–guest structure, causing a distorted equilibrium geometry of the isolated molecule compared to its geometry in the gas phase. This results in wavenumber shifts in the infrared spectrum and can cause band splitting. Usually, these effects can be empirically interpreted by comparing different host materials and modifying experimental parameters, such as temperature and host–guest mixing ratio, deposition speed, etc. We discuss the matrix effects observed for methanol in more detail in the SI and present our final assignment of the mid-IR spectrum of methanol as isolated in Ar and Ne in Figure 6. Table 3 lists the assignment of the 12 fundamental vibrations of methanol. It includes experimental MI-IR wavenumbers and calculated harmonic/anharmonic wavenumbers from the present study, compared to the gas-phase IR data by Serrallach et al. from 1974.^90^ The mean absolute deviation (MAD) between MI-IR and gas phase illustrates the matrix wavenumber shift ( = 3.3–4.4 cm^–1^ and  = 2.6–2.9 cm^–1^). As the VCI calculations do not include the matrix environment, we expect deviations between VCI and the MI-IR spectra. Hence, we determine the accuracy w.r.t. gas-phase IR data. The VCI calculations agree better with gas-phase IR data than the harmonic wavenumbers ( = 37.1–58.6 cm^–1^,  = 3.9–4.5 cm^–1^). Moreover, the VCI calculations show “spectroscopic accuracy” sufficient to assign all bands in the MI-IR spectrum.

**Table 3:** Usual Assignment of Methanol’s 12 Fundamental Vibrations, Highlighting Matrix Shifts for Argon () and Neon () as well as Computational Errors in Harmonic () and Anharmonic () Computations, when Compared to Gas-Phase IR Experiments^a^

Label		Matrix				Gas		Computation			
Spec.	Chem.	Ar		Ne		(a)	(b)	Harm		VCI	
**CH**_**3**_**OH**											
	νOH	3666.7	–14.8	3690.1	8.6	3681	3681.5	3886.6	205.1	3697.3	15.8
	*ν_s_CH_3_*	3006.4	7.4	2996.3	–2.7	3000	2999.0	3124.0	125.0	3005.7	6.7
b	*ν_as_CH_2_*	2961.8		2966.9		2960	2970 ± 4	3103.7		2960.6	
		2930.0		2928.2			2929.5			2930.9	
		2914.2		2912.0			2912 ± 4			2914.0	
	*ν_s_CH_3_*	2848.0	3.8	2841.5	–2.7	2844	2844.2	3031.4	187.2	2847.2	3.0
	*δ_s_CH_3_*	1473.5	–3.7	1477.0	–0.2	1477	1477.2	1518.6	41.4	1476.0	–1.2
	*δ_as_CH_2_*	1466.3	1.3	1469.0	4.0	1477	1465 ± 3	1516.3	51.3	1471.0	6.0
	*δ_s_CH_3_*	1451.8	–2.7	1450.5	–4.0	1455	1454.5	1485.5	31.0	1453.1	–1.4
		1329.6	–2.4	1331.4	–0.6	1345	1332.0	1366.5	34.5	1340.7	8.7
	*δ_as_CH_3_*	1162.8	17.8	1156.5^e^	11.5		1145 ± 4	1195.4	50.4	1154.8	9.8
	*	1076.3	1.8	1071.4	–3.1	1060	1074.5	1101.8	27.3	1072.2	–2.3
		1033.9	0.4	1032.6	–0.9	1033	1033.5	1042.3	8.8	1036.7	3.2
		271.5^c^				80–300		210.5		337.4	
MAD			4.3		2.9				58.6		4.5
**CH**_**3**_**OD**											
	*ν_s_CH_3_*	3006.9	5.9	2996.2	–4.8	3000	3001.0	3124.2	123.2	3010.5	9.5
b	*ν_as_CH_2_*	2955.3		2953.6		2960	2950 ± 2	3103.6		2959.5	
		2920.9		2920.7			2922.8			2930.1	
		2908.8		2905.5			2907.0			2910.4	
	*ν_s_CH_3_*	2845.5	4.7	2839.1	–1.7	2843	2840.8	3031.4	190.6	2844.2	3.4
		2704.8	–12.8	2721.7	4.1	2718	2717.6	2830.3	112.7	2736.0	18.4
	_3_	1473.1	–4.4	1473.4	–4.1	1473	1477.5	1517.5	40.0	1476.1	–1.4
	_2_	1466.2	3.2	1467.6	4.6	1473	1463 ± 4	1516.2	53.2	1470.8	7.8
	_3_	1450.0	–5.0	1448.1	–6.9	1456	1455.0	1485.4	30.4	1452.6	–2.4
	*	1227.3	2.8	1231.0 ± 2	6.5	1230	1224.5	1255.0	30.5	1227.1	2.6
	_3_	1141.5^c^	–0.5			1160	1142 ± 4	1195.3	53.3	1137.2	–4.8
		1042.8	2.5	1039.9	–0.4	1040	1040.3	1070.6	30.3	1045.3	5.0
	*δ_ip_OD*	864.9	0.9	864.1	0.1	864	864.0	861.8	–2.2	865.5	1.5
	*δ_oop_OD*							166.9		264.3	
MAD			3.3		2.6				51.3		4.4
**CD**_**3**_**OD**											
	OD	2705.3	–12.1	2723.0	5.6	2724	2717.4	2830.6	113.2	2728.2	10.8
	*ν_s_CD_2_*	2255.0	5.0	2247.3	–2.7	2260	2250 ± 2	2319.4	69.4	2254.2	4.2
b	*ν_as_CD_3_*	2218.9		2209.4		2228	2212.6	2302.2		2213.3	
		2145.0		2144.3							
	*ν_s_CD_3_*	2078.0	4.0	2072.7	–1.3	2080	2074.0	2171.1	97.1	2070.0	–4.0
	*δ_s_CD_3_*	1127.9	–5.6	1129.0	–4.5	1135	1133.5	1158.3	24.8	1130.5	–3.0
	*δ_s_CD_3_*	1068.2	–9.8	1069.0	–9.0	1080	1078 ± 2	1103.8	25.8	1077.6	–0.5
	*δ_as_CD_2_*	1064.1	–5.2	1062.1	–7.2	1060	1069.3	1095.6	26.3	1072.9	3.6
	*	1031.5^c^	3.7	1028.7	0.9	1024	1027.8	1069.3	41.5	1042.9	15.1
		983.0	3.0	979.9	–0.1	983	980.0	994.5	14.5	983.3	3.3
	*δ_as_CD_3_*	895.0^c^	3.0	890.3	–1.7	892^d^	892	922.4	30.4	894.0	2.0
	*δ_ip_OD*	775.9	1.1	774.4	–0.4	776	774.8	772.2	–2.6	774.2	–0.6
	*δ_oop_OD*							153.2		231.6	
MAD			4.4		2.8				37.1		3.9

a Neon and argon MI-IR data from this study compared to gas-phase IR assignments from (a) pure gas-phase experiments (1972, Shimanouchi et al.^80^) and (b) as influenced by matrix-isolation experiments (1974, Serralach et al.^90^ Deviations are w.r.t. to assignment in (b). The VCI computation setup is discussed in Dinu et al.^28^

b This resonance cannot be assigned uniquely (c.f. Figure 7).

c Taken from ref (90).

d Taken from ref (85).

e Taken from ref (123).

### 2.3 Assignment of Resonances in the Mid-IR Spectrum of Methanol

The assignment in Table 3 is naive because it only considers the fundamental vibrations, which is insufficient to describe resonances. For example, the VCI calculation yields three transitions that have contributions from the  fundamental in CH_3_OH (2960.6, 2930.9, and 2914.0 cm^–1^). A naive assignment chooses a band in the experiment that is close to the calculated value of 2960.6 cm^–1^ to uniquely assign it to the  fundamental. In a correct assignment, one must consider resonances, and the assignment becomes ambiguous. To discuss the issue of ambiguous assignments, let us first recapitulate some central observations for the MI-IR spectrum of CH_3_OH:

(1) There are multiple bands within 1480–1450 cm^–1^ and 2960–2900 cm^–1^. It is not possible to explain all observed bands with the few fundamentals predicted in the harmonic approximation: three *CH_3_* fundamentals ν_4_, ν_5_, ν_10_ within 1480–1450 cm^–1^ and one  fundamental ν_9_ within 2960–2900 cm^–1^.
(2) The region of 2960–2900 cm^–1^ is twice in wavenumber compared to the 1480–1450 cm^–1^ region. The overtones and combination bands of the *CH_3_* fundamentals within 1480–1450 cm^–1^ could be assumed to contribute to 2960–2900 cm^–1^. Thus, one may assign all bands within 2960–2900 cm^–1^ using the labels 2ν_4_, 2ν_10_, 2ν_5_ for overtones and ν_4_+ν_10_, ν_5_+ν_10_, and ν_5_+ν_4_ for combination bands.
(3) In both regions, the observed bands are close in wavenumber. Hence, the underlying fundamentals, overtones, and combinations in the theoretical model should also be similar in energy. We may denote such energetically close vibrations as “quasi-degenerate” (cf. Fermi resonance^27^ in CO_2_ or Darling-Dennison resonance^47^ in H_2_O).

In other words, we (1) cannot assign all bands based on the notations derived within the harmonic approximation. Thus, we (2) extend the notation by introducing overtones and combinations. As the bands are close in energy, we (3) introduce resonances to explain the interplay of fundamentals, overtones, and combination bands. This interpretation is precisely what Fermi has proposed for CO_2_. As resonances include contributions from multiple quasi-degenerate vibrations, an unambiguous assignment of resonances by labeling specific experimental bands within the conventional notations is impossible. Any attempt to do so will end in a stalemate, resulting in ambiguous assignments.

#### 2.3.1 Assignment via Deuteration

As Bartholome showed in 1935 for methanol in gas-phase, ambiguous assignments can become tangible by isotopic substitution.^175^ Consider the νCH region (2960–2900 cm^–1^) and the δCH region (1480–1450 cm^–1^). As these regions can be primarily attributed to the CH_3_ group, we expect that *hydroxyl deuteration* (OH to OD) will only faintly change the spectral patterns (cf. Bouteiller and Perchard^51^), while *methyl deuteration* (CH_3_ to CD_3_) should introduce significant changes. Our MI-IR spectra mostly fulfill these expectations (cf. Figure 6).

Hydroxyl deuteration from CH_3_OH to CH_3_OD slightly changes the overall shape of the bands in the νCH region. For CH_3_OH, the bands are rather broad and of similar weak intensity. For CH_3_OD, pronounced peaks can be distinguished. However, in Ar and Ne matrices, both CH_3_OH and CH_3_OD show complex spectral patterns with roughly four to six bands in a spectral range of 2960–2900 cm^–1^.

Upon methyl deuteration to CD_3_OD, the νCD region (2300–2100 cm^–1^) is three times wider than the corresponding νCH region, and the δCD region (1150–1050 cm^–1^) is five times wider than the corresponding δCH region. In our MI-IR spectra, we observe for CD_3_OD that the number of bands and the splitting drastically reduces in the νCD region compared to the νCH region, and in the δCD region, we observe well-separated bands.

As stated above, the assignment in the νCH region of CH_3_OH and CH_3_OD is partially “ambiguous.″ However, the assignment of the νCD region in CD_3_OD is straightforward. We could map the assignment from the deuterated species to the non-deuterated species, assuming that the species’ molecular vibration behaves similarly. While this workaround is suitable, it neglects the resonances necessary to explain the complex spectrum in the non-deuterated species.

#### 2.3.2 Assignment with Resonances

In their Raman experiments of CH_3_OH from 2013, Yu et al. state that “the spectral assignments in the C–H stretching region tend to be ambiguous”.^176^ They based their assignment on calculations using scaled harmonic wavenumbers from density functional theory, which do not explicitly account for overtones, combination bands, and resonances. A better-suited approach was presented in 2000 by Miani et al., who have calculated the Fermi resonances for various deuterated methanol molecules^50^ using perturbation theory. Following this approach, Bouteiller and Perchard provided a first rigorous assignment of resonances for N_2_ matrix isolation IR experiments considering the CH stretching region of CH_3_OD (cf. P2 polyad in ref (51)). Compared to Miani et al., they also include Darling-Dennison resonances based on perturbation theory.

These studies demonstrate that an anharmonic approach is necessary to include a correct description of resonances. However, using perturbation theory can be problematic, as specific transitions may be neglected to not cause unreasonable denominators in the perturbation terms.^51^ Variational approaches like the present VCI calculations do not suffer from these problems. Thus, we can reconsider our naive assignments from Table 3 and perform a resonance analysis from VCI calculations. Here, we limit our discussion to selected regions of the Ne MI-IR spectra. We refer to the Supporting Information for more details on the complete mid-IR spectra in Ne and Ar matrices.

(a) For CH_3_OH, we assign the **symmetric stretch ν**_*s*_**CH**_**3**_, or , to 2841.5 cm^–1^ in Ne matrix. Our VCI calculation yields a state at 2847.2 cm^–1^ composed of the configurations . In other words, the configuration in which the normal-mode *q*_9_ is singly excited has a contribution of 51.0%, and the configuration in which the normal-mode *q*_6_ is doubly excited has a contribution of 26.2%. In the spectroscopist notation, the VCI state at 2847.2 cm^–1^ has contributions from ν_3_ (51.0%) and 2ν_5_ (26.2%).

We may conclude that our initial assignment for CH_3_OH is reasonable because the configuration labeled as ν_3_ has the highest contribution. To highlight that the overtone 2ν_5_ is involved, we could denote this as a “Fermi resonance,″ indicating the interaction between a fundamental and an overtone. However, this notation is not complete. As can be seen in the Sankey diagram in Figure 7a, our VCI calculations suggest that the overtone 2ν_5_ contributes to two other VCI states (2922 and 2928 cm^–1^). Both of these states have further contributions from other configurations.

**Figure 7:**
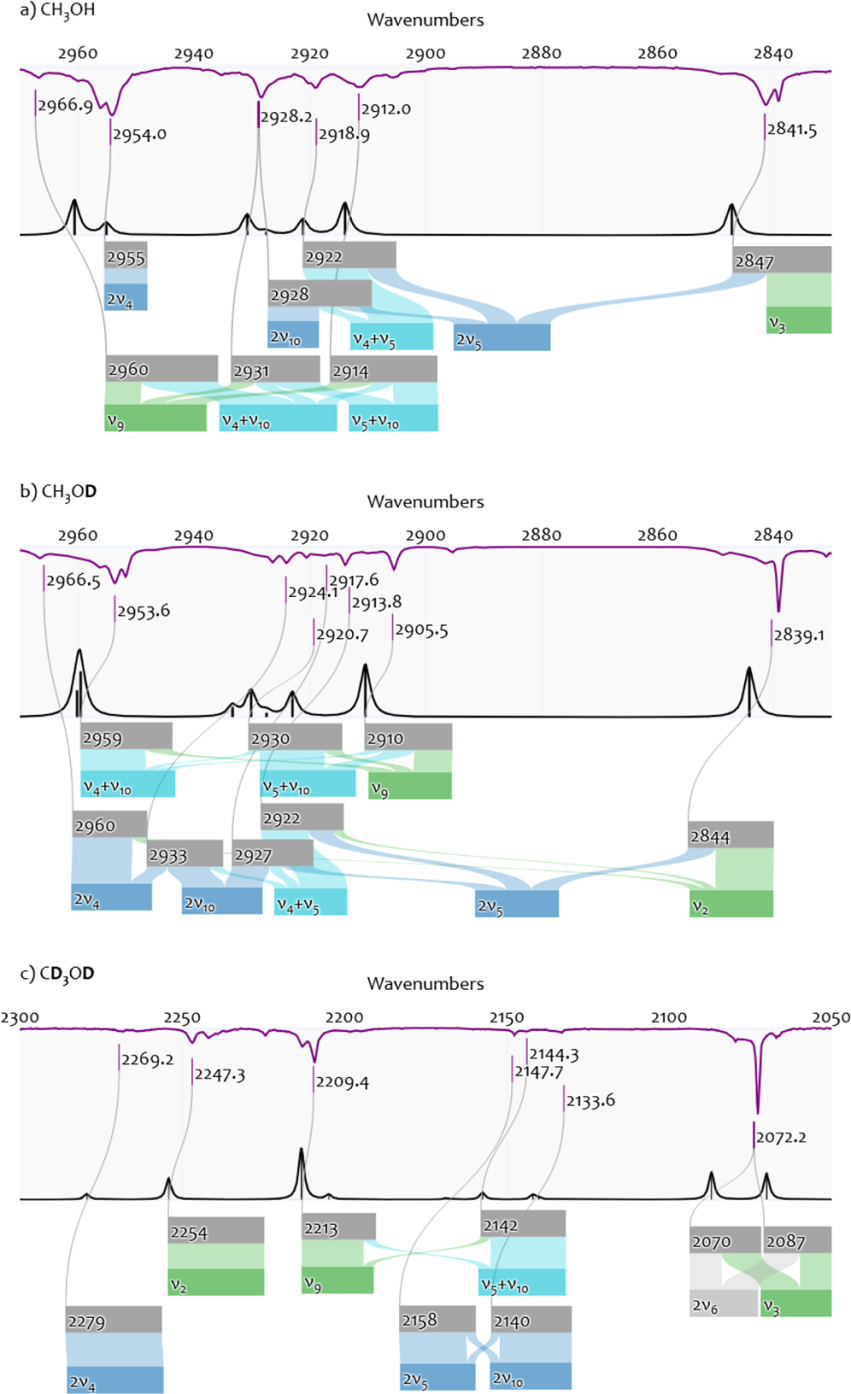
Resonances in the Ne matrix-isolation IR spectrum of a) CH_3_OH, b) CH_3_OD and c) CD_3_OD. The upper part of each panel shows an MI-IR spectrum on the top (purple) and a VCI spectrum on the bottom axis (black). The lower part shows the resonance analysis as Sankey diagrams, illustrating the contributions from VCI configurations (colored boxes) to the VCI wavenumbers (gray boxes) and their link to the experimentally observed wavenumbers (purple). VCI configurations are labeled by the spectroscopic notation and colored by the chemist notation (cf. Figure 4). Further details are in Tables S3-S51.

For CH_3_OD, the resonance pattern is similarly complicated. Initially, we assigned the fundamental ν_*s*_CH_3_ or  to 2839.1 cm^–1^ in Ne matrix. VCI yields a state at 2844.2 cm^–1^ with contributions from ν_2_ (57.2%) and 2ν_5_ (27.4%). Again, our initial assignment is reasonable because the configuration labeled ν_2_ has the highest contribution. However, as can be seen from the Sankey diagram in Figure 7b, our VCI calculation suggests that both the fundamental ν_2_ and the overtone 2ν_5_ are also involved in each two VCI states (ν_2_: 2960 and 2922 cm^–1^, 2ν_5_: 2927 and 2922 cm^–1^),

For CD_3_OD, our VCI calculation suggests a resonance of two states (2087 and 2070 cm^–1^) with almost equal contributions from ν_3_ and 2ν_6_ (note that the normal mode behind the label ν_6_ corresponds to a delocalized motion). As shown in Figure 7c, our VCI calculation does not imply further resonances with these two states. The assignment of the fully deuterated species is, thus, less ambiguous. Our MI-IR spectra show some bands in this region that could be associated with this resonance. However, due to the broadness of the bands, we avoid a final assignment of both states and merely assign the fundamental  to 2072.2 cm^–1^ in the Ne matrix.

(b) For CH_3_OH, we assign the **antisymmetric stretch ν**_*as*_**CH**_**2**_, or , to 2966.9 cm^–1^ in Ne matrix, in analogy to historically established assignments.^90^ VCI yields a state at 2960.8 cm^–1^, with a contribution of 59.9% from the combination band , compared to only 28.3% from the fundamental ν_9_. This suggests labeling this state as  rather than ν_9_ and contradicts the established assignment. Considering at least one other VCI state in which  and ν_9_ are involved, the assignment becomes more feasible. VCI provides two candidates, one at 2930.9 cm^–1^ with contributions from  (57.6%),  (14.3%) and ν_9_ (19.7%), and another one at 2914.0 cm^–1^ with contributions from  (35.0%),  (16.2%) and ν_9_ (33.0%) (cf. Figure 7).

In the Ne MI-IR experiments, we can reasonably assign the resonance pattern between the fundamental ν_9_ and the two combination bands , and  to three bands: 2966.9, 2928.2, and 2912.0 cm^–1^. If we adhere to unique assignments preferring the highest contributing configuration, we label the band at 2966.9 cm^–1^ as  and the band at 2912.0 cm^–1^ as . In this case, the fundamental ν_9_ would not occur in our assignment. This is somewhat misleading as the ν_9_ configuration has non-negligible contributions to both states, according to the VCI analysis. Hence, mentioning the resonance is, in any case, preferable and may be represented as shown in Figure 7a.

Considering the resonance pattern, similar statements can be made for CH_3_OD, as seen from Figure 7b. For CD_3_OD, the resonance becomes simpler. While our VCI calculations still suggest resonances, the contributions from the individual configurations are more pronounced (2213.3 cm^–1^ is 64.4% ν_9_, 2142.0 cm^–1^ is 78.0% ). This makes the unique assignment more reasonable compared to the nondeuterated species.

(c) So far, we have discussed that (a) for the symmetric stretch ν_*s*_CH_3_ region the overtone 2ν_5_ is involved in resonance with the fundamental ν_3_ and (b) for antisymmetric stretch ν_*as*_CH_2_ region the combination bands  and  are involved in resonance with the fundamental ν_9_. We observe for both above-mentioned cases (a) and (b) that deuteration of the methyl group, i.e., for CD_3_OD, makes the resonances less complicated. As shown from the Sankey diagrams in Figure 7, further **overtones and combinations** are involved in resonances. Again, deuteration tends to simplify the number of contributions.

For CH_3_OH, the combination  is involved in a resonance around the fundamental ν_3_, which includes two further overtones (2ν_10_, 2ν_5_). For CH_3_OD,  is part of a similar resonance pattern including three further overtones (2ν_4_, 2ν_10_, 2ν_5_). For CD_3_OD, the  combination has no pronounced contribution to any resonance.

Finally, we discuss the bending vibrations’ overtones (2ν_4_, 2ν_10_, 2ν_5_). Our VCI calculations for CH_3_OH (cf. Figure 7a) suggest a unique assignment for 2ν_4_ (2955 cm^–1^), while 2ν_10_ and 2ν_5_ are involved in a resonance of three states (2928, 2922, 2847 cm^–1^). For CH_3_OD (cf. Figure 7b), all three overtones are involved in a distributed resonance pattern (2960, 2933, 2927, 2922, 2844 cm^–1^), however, with a tentative unique assignment for 2ν_4_ (2960 cm^–1^). Only in CD_3_OD (cf. Figure 7c) can we assign all three overtones uniquely with the VCI calculations for 2ν_4_ (2279 cm^–1^), 2ν_5_ (2158 cm^–1^), and 2ν_10_ (2140 cm^–1^).

## 3 Conclusion

Despite being studied for a century, the vibrational spectrum of methanol still contains bands of unclear origin. This is due to the limited understanding of resonances and the use of conventional notations, which are insufficient to describe mode coupling and resonances adequately. In the present work, we define these limits and clarify them based on a combination of experiment and *ab initio* calculations.

Regarding matrix-isolation infrared (MI-IR) experiments, the presented vibrational self-consistent field and configuration interaction (VSCF/VCI) calculations can be considered to provide spectroscopic accuracy, i.e., the deviation between calculated and experimental transitions (4 cm^–1^, cf. Table 3) is smaller than the distance between observed transitions. Only vibrational transitions must be distinguished because rotation is quenched in MI-IR spectra. Compared to previous studies on MI-IR spectra combined with vibrational perturbation theory (VPT), where similar accuracy was achieved,^51^ the present VSCF/VCI calculations are variational, i.e., they do not need parametrization and obtain resonances as a “natural” result.

In agreement with the early MI-IR studies on methanol,^90^ we demonstrate that MI-IR spectroscopy resolves the fundamental vibrational transitions complementary to gas-phase IR spectroscopy. However, in contrast to these early studies, today, calculations provide a much better interpretation of the experiment.^51^ Our VSCF/VCI calculations put us into a position where we can complete the assignment of molecular vibration from MI-IR spectra in the mid-IR region of CH_3_OH, CH_3_OD, CD_3_OD (cf. Figure 6), by considering fundamentals, overtones, combination bands, and resonances. Furthermore, the calculations agree with the experiment showing that the number of resonances decreases with deuteration. The present calculations correctly interpret the mid-IR spectrum. The large amplitude motion of torsion is better described with a Hamiltonian in curvilinear coordinates.^40,155^

Our combined experimental and theoretical methodology allows us to reconsider the capabilities of conventional vibrational notations (cf. Figure 4 and Table 1). We show how the chemist and spectroscopist notations fail to describe many features in the spectrum. This can lead to problematic uses of the “resonance” term, most often by incorrectly assigning only parts of resonances and assuming these as unique. We plead for communicating all contributions to the resonances, as done by physicist notations. As this notation is *per se* not descriptive, we suggest reasonably mapping the physicist notation to the more descriptive spectroscopic and chemist notations. A recipe for such a two-layered notation is based on three aspects:

1. The calculated anharmonic wavenumbers should quantitatively agree with an experimental observation. [Here, we use VSCF/VCI calculations to obtain the anharmonic wavenumbers, which are accurate for assigning pure vibrational transitions in MI-IR experiments.]
2. The basis for expanding the anharmonic theoretical model should be descriptive. [In the present VSCF/VCI calculations, normal modes are the coordinates for the basis in which the wave function is expanded. The normal modes are descriptive in terms of conventional vibrational notations, and this quality is maintained throughout the VSCF/VCI calculation.]
3. There should be a possibility to calculate contributions from the basis (2) to the anharmonic wavenumber (1). [In the VCI approach, the wave function is a linear combination of configurations, i.e., a weighted sum. The weights of this sum reflect contributions.]

We may conclude with an example from the present study on methanol, particularly the resonance between the ν_9_ fundamental and the  combination. One of the corresponding experimentally observed bands in the spectrum is usually uniquely assigned to the ν_9_ fundamental to facilitate communication. However, this neglects the importance of the  combination and, worst case, oversees the assignment of another experimentally observed band which is part of the resonance. We cannot assign one without the other in a correct assignment and must use a two-layered notation, as mentioned above.

VPT or VSCF/VCI calculations are common routes for obtaining the necessary numerical quantities for such a two-layered notation. However, these are not black-box procedures; resonance analyses must be carefully checked for every molecule. For high-resolution spectroscopy, even more sophisticated calculations may be necessary. However, the general issue of ambiguity in the notation remains. While these aspects are known in the research field of molecular spectroscopy, the representation of two-layered notations tends to be an obstacle when other research fields adopt spectroscopic results.

We propose considering Sankey diagrams for illustrating a two-layered notation (cf. Figure 7). Although these diagrams have theoretical considerations, their interpretation should be accessible to a broad audience. The diagram conveys an intricate assignment and helps to evaluate whether certain spectral features can function as marker bands for further studies, e.g., for observational purposes in atmospheric chemistry or astrochemistry or the investigation of chemical reaction mechanisms via IR spectroscopy. We have recently demonstrated the applicability of such Sankey diagrams for improving the assignment of matrix-isolated carbonic acid.^177^ Nevertheless, the benefits of such diagrams for more complex resonance patterns, as studied in high-resolution spectroscopy, remain to be evaluated.

## 4 Methodology

This study’s matrix-isolation (MI) setup was previously described.^28^ We prepare host–guest mixtures using a pressure gauge. A 1:500 mixture corresponds to 2 mbar of the guest diluted with about 990–1010 mbar. We deposit the mixtures with a constant flow of 4 mbar/min from a volume of about 200 mL and a pressure of 900–980 mbar. The deposition time is about 45 min per layer. This corresponds to 190 mbar per layer, which is for a volume of 200 mL, around 1.47 mmol of substance. The gas is deposited onto a gold-plated mirror in the high-vacuum cryostat chamber at 10^–7^ mbar and a temperature of 5.8 K. Before each sample measurement, we take a background spectrum. We accumulate 512 scans at a resolution of 0.3 cm^–1^.

The calculations rely on the Molpro software package (version 2023).^168,178^ With the normal-modes *q*_*i*_ as a coordinate system, the SURF algorithm constructs a N-mode potential energy surface (PES), as presented by Rauhut et al.^42,171^ For the PES construction, we considered different setups in the choice of electronic structure theory.^28^ The PES is initially constructed on a grid for CH_3_OH and transformed to the CH_3_OD and CD_3_OD using the PESTRANS algorithm.^170^ The isotopic transformation relies on an analytical representation of the PES obtained from the POLY algorithm.^169^ Based on the analytical PES representation, the vibrational self-consistent field (VSCF) approach solves the vibrational Schröodinger eq. Vibration configuration interaction (VCI) for each VSCF vibrational state specifically accounts for correlation. The configurations for the VCI computation are excitations from the VSCF reference. The number of simultaneous excitations is limited to quadruples VCI(4) when using a 3-mode PES (quintuples VCI(5) when using a 4-mode PES). Each mode is limited to a maximum excitation of 5, while the total excitation level is 12 (15 when 4-mode PES are employed). Although this truncation significantly limits the VCI space, it is further reduced by an iterative configuration-selective scheme to lower computational costs.^45^

The Sankey diagrams have been created using the Plotly.^179^ HTML versions of the Sankey diagrams are available at https://lab.dedin.eu.

## Supporting Information Available

Experimental methodology and computational setup, details on the vibrational notations of methanol, a complete description of the methanol’s argon and neon MI-IR spectra assignment, and a complete description of the resonances (PDF)
