# Supplementary material for: How Vibrational Notations Can Spoil Infrared Spectroscopy: A Case Study on Isolated Methanol
Source: ACS Phys Chem Au. 2024 Oct 4;4(6):679–95. doi: 10.1021/acsphyschemau.4c00053 (PMC11613289; doi:10.1021/acsphyschemau.4c00053)
Supplement: Supplementary file 1 — pg4c00053_si_001.pdf [file pg4c00053_si_001.pdf]

How vibrational notations can spoil infrared spectroscopy: a case study on isolated methanol

Supplementary Information

Dennis F. Dinu*,†,‡,¶ Kemal Oenen,‡ Jonas Schlagin,‡ Maren Podewitz,† Hinrich Grothe,† Thomas Loerting,¶ and Klaus R. Liedl‡

†Institute of Materials Chemistry, TU Wien, Getreidemarkt 9/165 1060 Vienna, Austria
‡Department of General, Inorganic and Theoretical Chemistry, University of Innsbruck, Innrain 80, 6020 Innsbruck, Austria
¶Department of Physical Chemistry, University of Innsbruck, Innrain 52, 6020 Innsbruck, Austria

* Dennis.Dinu@uibk.ac.at

Contents

MI-IR spectrum and VCI calculations of carbon dioxide (CO_2) 2
Vibrational notations 2
MI-IR spectrum and VCI calculations of methanol (CH_3OH, CH_3OD and CD_3OD) 5
Oligomerization, matrix effects, torsion-vibration splitting 6
Region (I) - The *νCO* and *δOH* region (ν_6, ν_7, ν_8) 7
Region (II) - The *δ*CH_3 region (ν_4, ν_5, ν_{10}, ν_{11}) 8
Region (III) - The *νOH* and the "unambiguous" stretch region 9
Region (IV) - The "ambiguous" stretch region 11
Supplementary References 16

MI-IR spectrum and VCI calculations of carbon dioxide (CO_2)

We elaborate on the assignment of the MI-IR spectrum of carbon dioxide, shown in **Table S2**, in a previous publication (S*2*).

Table S1: Assignment of the carbon dioxide IR spectra based on VCI analysis.

	**Experiment** [a)]			**Computation** [b)]	
	Ar	Ne	Gas	$\nu^{VCI(5)}$	configuration (contribution)
$^{12}C^{16}O_2$	3707.8 / 3700.2	3714.7	*3715.6*	3713.8	$\nu_3 + 2\nu_2$ (51.4 %), $\nu_3 + \nu_1$ (44.9%)
	3603.8 / 3597.6	3612.4	*3613.7*	3610.6	$\nu_3 + 2\nu_2$ (47.8 %), $\nu_3 + \nu_1$ (49.2%)
	2345.1 / 2339.6	2348.2	*2349.9*	2347.4	ν_3
	-	-	*1388.2*	1388.1	$2\nu_2$ (54.2 %), ν_1 (45.2%)
	-	-	*1285.5*	1284.6	$2\nu_2$ (45.0%), ν_1 (54.5%)
	663.7 / 662.1	668.5 / 667.9	*668.2*	667.8	ν_2

a) Experimental Ne and Ar MI-IR spectra from ref. (S*2*). Gas-phase IR data from ref. (S*3*) and Raman data from ref. (S*4*)
b) VCI(5) wavenumbers computed using a multi-mode PES at CCSD(T)-F12/VTZ-F12 level of theory (S*2*)

Vibrational notations

The conventional vibrational notations are shown in **Table 1** and visualized in **Figure 4** in the main article. The differences of the notations for the isotopologues are shown in **Figure 5** in the main article. Here, we additionally highlight these differences by including the normal mode vectors in **Table S2**. The chemist notation must be appreciated with care, as it relies on a qualitative localization of normal modes by identifying principal motion patterns. As highlighted in **Table 1**, we achieve this in two ways. We visualize the nuclear movement along the normal modes and qualitatively decide which part of the molecule moves the most. This somewhat arbitrary approach leads to contradictory labels depending on human decision-making. Thus, we also decompose the normal mode into internal coordinates, yielding a numerical interpretation (S*1*). **Figure S1** depicts the normal mode decomposition via contribution tables. We show how much a primitive internal coordinate contributes to a normal mode.

Using a specific label from the chemist notation is not unique for all normal modes. The most striking example is *νCO*. We use this label for two normal modes, namely q_3 (1107 cm^{-1}) and q_2 (1046 cm^{-1}) in CH_3OH, q_5 and q_3 in CH_3OD, and q_5 and q_4 in CD_3OD. Both normal modes resemble a rather complicated vibration of the complete methanol scaffold. In this scaffold vibration, one principal motion pattern is the vibration between the C and the O atoms. Thus, we may label both normal modes as *νCO*. Extending the chemist's notation using more than one principal motion pattern as a label can further distinguish these two normal modes. Although this procedure may be successful for the discussion of methanol, it is not extendable for larger systems, where scaffold vibrations are so complex that they cannot be distinguished anymore in the chemist's notation. For the sake of simplicity, we remain vague in labeling those two normal modes by giving them the same *νCO* label. Note that the normal mode decomposition in **Figure S1** reflects this issue very well.

Table S2. Normal mode vectors and conventional notations methanol (CH_3OH, CH_3OD, and CD_3OD).
Spectroscopist notation ← isotopologue normal mode, numbered after sort by their harmonic wavenumbers (cf. Table 1).

CH_3OH	CH_3OD	CD_3OD
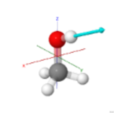 νOH<br>**ν_1 (A')** ← q_{12} (A')	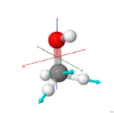 $\nu_s CH_3$<br>**ν_1 (A')** ← q_{12} (A')	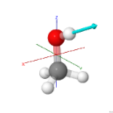 νOD<br>**ν_1 (A')** ← q_{12} (A')
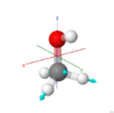 $\nu_s CH_3$<br>**ν_2 (A')** ← q_{11} (A')	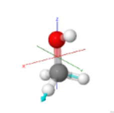 $\nu_{as} CH_2$<br>**ν_9 (A'')** ← q_{11} (A'')	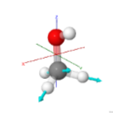 $\nu_s CD_3$<br>**ν_2 (A')** ← q_{11} (A')
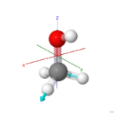 $\nu_{as} CH_2$<br>**ν_9 (A'')** ← q_{10} (A'')	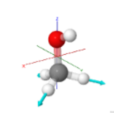 $\nu_s CH_3$<br>**ν_2(A')** ← q_{10} (A')	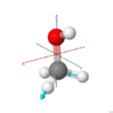 $\nu_{as} CD_2$<br>**ν_9 (A'')** ← q_{10} (A'')
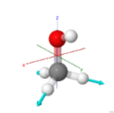 $\nu_s CH_3$<br>**ν_3 (A')** ← q_9 (A')	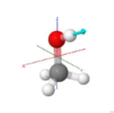 νOD<br>**ν_3 (A')** ← q_9 (A')	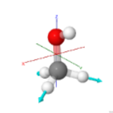 $\nu_s CD_3$<br>**ν_3 (A')** ← q_9 (A')
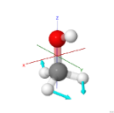 $\delta_s CH_3$ (scissor)<br>**ν_4 (A')** ← q_8 (A')	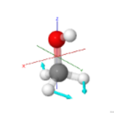 $\delta_s CH_3$ (scissor)<br>**ν_4 (A')** ← q_8 (A')	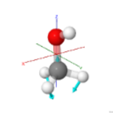 $\delta_s CD_3$ (umbrella)<br>**ν_4 (A')** ← q_8 (A')
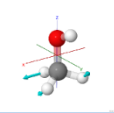 $\delta_{as} CH_3$ (wagging)<br>**ν_{10} (A'')** ← q_7 (A'')	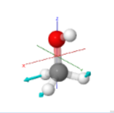 $\delta_{as} CH_3$ (wagging)<br>**ν_{10} (A'')** ← q_7 (A'')	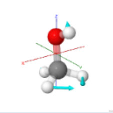 $\delta_s CD_3$ (scissor)<br>**ν_5 (A')** ← q_7 (A')
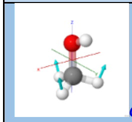 $\delta_s CH_3$ (umbrella)<br>**ν_5 (A')** ← q_6 (A')	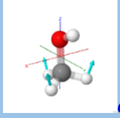 $\delta_s CH_3$ (umbrella)<br>**ν_5 (A')** ← q_6 (A')	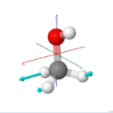 $\delta_{as} CD_3$ (wagging)<br>**ν_{10} (A'')** ← q_6 (A'')
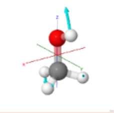 δ_{ip}OH<br>**ν_6 (A')** ← q_5 (A')	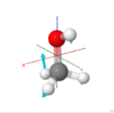 νCO + $\delta_s CH_3$ (rock) + δ_{ip}OD<br>**ν_6 (A')** ← q_5 (A')	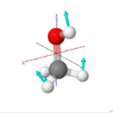 νCO + $\delta_s CD_3$ (rock) + δ_{ip}OD<br>**ν_6 (A')** ← q_5 (A')
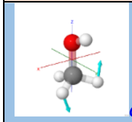 $\delta_{as} CH_3$ (rock)<br>**ν_{11} (A'')** ← q_4 (A'')	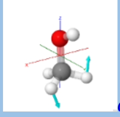 $\delta_{as} CH_3$<br>**ν_{11} (A'')** ← q_4 (A'')	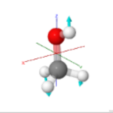 νCO<br>**ν_7 (A')** ← q_4 (A')
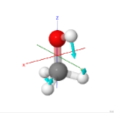 νCO + $\delta_s CH_3$ (rock) + δ_{ip}OH<br>**ν_7 (A')** ← q_3 (A')	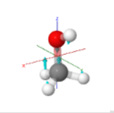 νCO<br>**ν_7 (A')** ← q_3 (A')	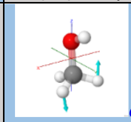 $\delta_{as} CD_3$<br>**ν_{11} (A'')** ← q_3 (A'')
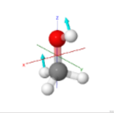 νCO<br>**ν_8 (A')** ← q_2 (A')	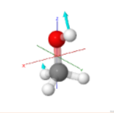 δ_{ip}OD<br>**ν_8 (A')** ← q_2 (A')	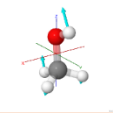 δ_{ip}OD<br>**ν_8 (A')** ← q_2 (A')
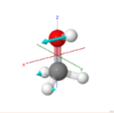 δ_{oop}OH<br>**ν_{12} (A")** ← q_1 (A")	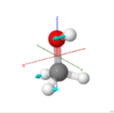 δ_{oop}OD<br>**ν_{12} (A")** ← q_1 (A")	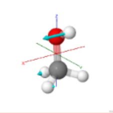 δ_{oop}OD<br>**ν_{12} (A")** ← q_1 (A")

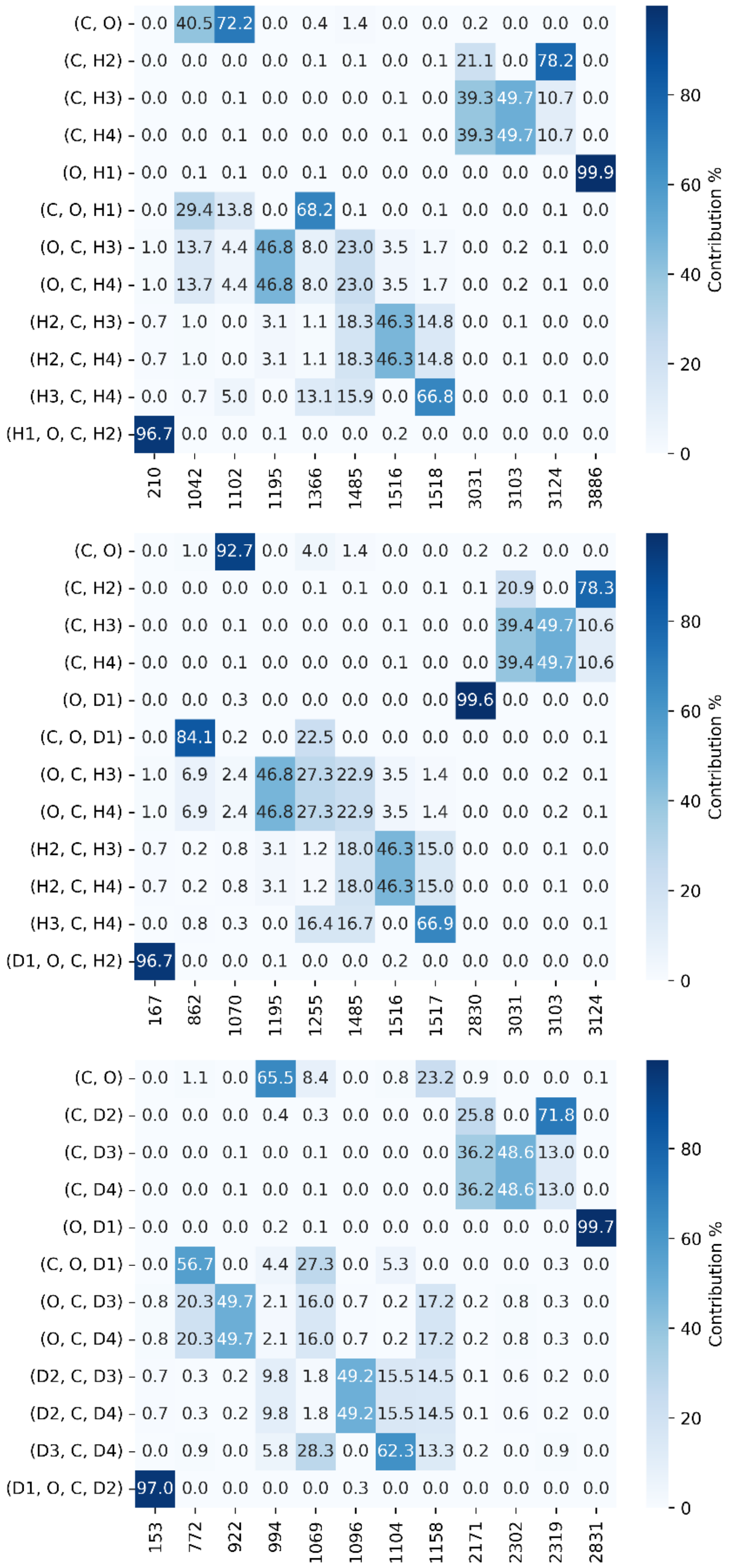

Figure S1. Normal mode decomposition of methanol showing the contribution of an internal coordinate (y-axis) to a normal mode (x-axis) for CH_3OH (top), of CH_3OD (middle), and CD_3OD (bottom).

MI-IR spectrum and VCI calculations of methanol (CH_3OH, CH_3OD and CD_3OD)

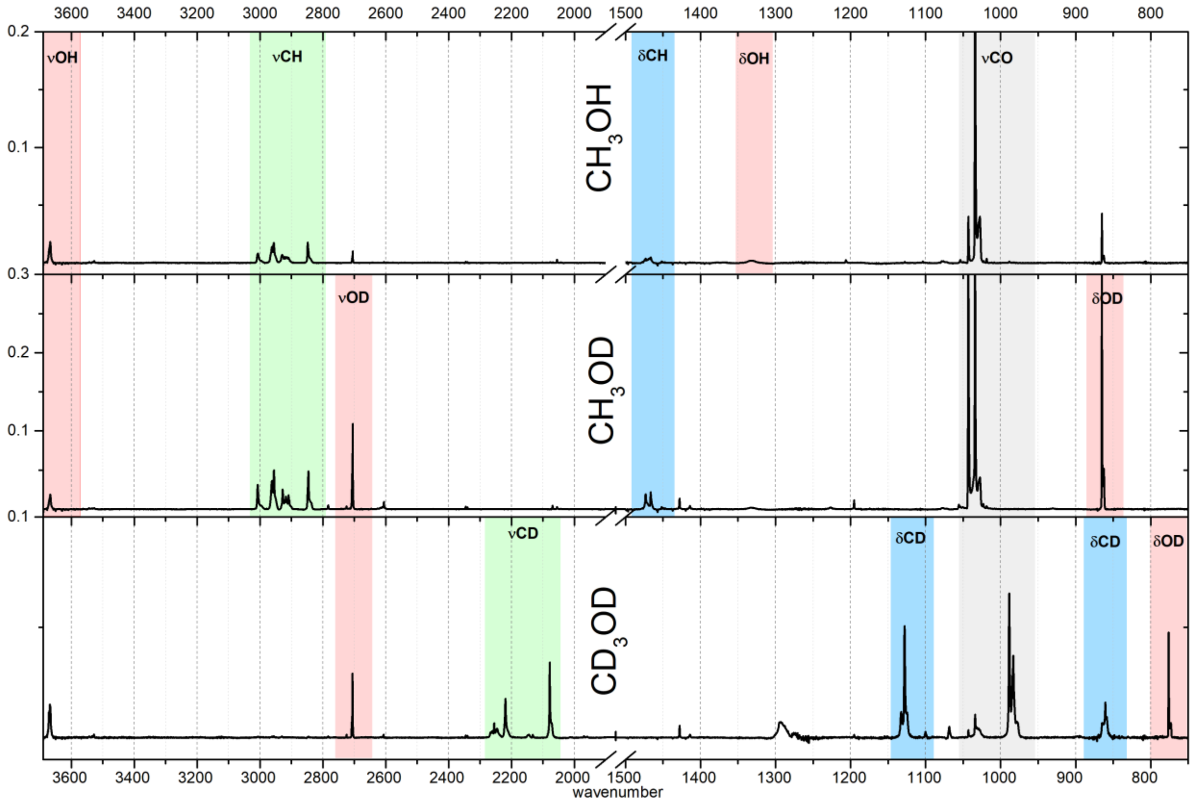

Figure S2. Wavenumber shifts due to deuteration, illustrated at the example of methanol isolated in Argon matrix in a mixing ratio of 1:500. The regions are colored based on the chemist notation (*e.g.*, red in the *νOH* region).

A first evaluation of the different isotopologues shows how the respective regions shift due to deuteration (cf. **Figure S2**). **Figure 6** in the article shows the MI-IR spectra of CH_3OH, CH_3OD, and CD_3OD, the upper three in Argon matrices and the lower three in Neon matrices. We slice the spectra into windows, focusing solely on methanol's spectral features. We color the spectra based on the chemist notation (*e.g.*, red in the *νOH* region). Furthermore, we label the spectral windows relying on the spectroscopist notation (e.g., $\nu_1(A')$ for the window comprising the *νOH* region). To discuss conventional vibrational notations in the IR spectrum of methanol, we distinguish the spectrum into four parts:

(I) The *νCO* region (black) and *δOH* region (red) with the ν_6, ν_7, and ν_8 vibrations.
(II) The δCH_3 region (blue) with the ν_4, ν_5, ν_{10}, and ν_{11} vibrations.
(III) The *νOH* region (red) and the unambiguous part of the *νCH* region (green) with the ν_1, ν_2, and ν_3 vibrations.
(IV) The ambiguous *νCH* region (cyan) with the ν_9 vibration.

The assignment in regions I – III is straightforward but tedious. Thus, we mention these regions only here in the supplementary information. We cannot maintain unambiguous assignments for Region IV and must consider multiple resonances. We discuss this in more detail in the main article. However, we first discuss matrix effects and methanol's characteristic torsion tunneling splitting before assigning the pure vibrational features of single methanol molecules in these regions.

Oligomerization, matrix effects, torsion-vibration splitting

For all isotopologues, we observe *oligomerization* by some bands that are redshifted by roughly 100 cm^{-1} w.r.t. the *vOH* fundamental of the monomer, which is well-known in literature (S*5*–S*11*). In the Ar spectra, these oligomer bands are observed with relatively low intensity for dilutions of 1:250 and 1:500 and are almost invisible for dilutions of 1:1000. In the Ne spectra, oligomer bands show 10-fold higher intensities than the monomer bands, even for the dilution of 1:1000. That means oligomerization is much more present in Ne matrices than in Ar matrices. Moreover, in the Ne spectra, we do not observe the monomer's *vOH* fundamental exclusively, even in the highest dilution experiment. We can widely rule out oligomerization in all other spectral regions than the vOH region.

It is well-known from previous studies that methanol does not rotate within the matrix (S*6*, S*12*). The matrix-isolation quenches *rotational-vibrational transitions*. However, we observe various splitting patterns in the *vOH* and *vCO* regions in both Ar and Ne spectra. As we rule out oligomerization or rotation as the reason for this splitting, the first naive assumption is that different matrix trapping sites occur. Considering the Ar matrices, assuming at least two trapping sites is viable.

In contrast to Ar matrices, there are probably no different trapping sites for methanol in Ne matrices. The complicated splitting patterns of methanol in Ne spectra arise from torsion-vibration tunneling splitting. Early gas-phase IR experiments of methanol suggest a hindered hydroxyl-group torsion, and estimates of the torsion barrier date back to the 1940s and 50s (S*13*, S*14*). The hindered internal torsion originates from *torsion-vibration tunneling,* resulting in splitting patterns in the corresponding spectrum. This splitting has been a central aspect of the gas-phase spectra assignment, to mention some early studies from the 1970s (S*15*, S*16*).

Today, there is a vast amount of literature on methanol in the gas phase, considering both the complex rotation-vibration interaction and torsion-vibration tunneling. We may refer to a recent gas-phase study by Xu et al. (S*17*). Recently, Lee et al. (S*18*) identified patterns in the para-H_2 MI-IR spectrum as torsion-vibration tunneling splitting, demonstrating a not completely hindered torsion for methanol in para-H_2 matrices. Perchard et al. reported similar splittings in Ne spectra and studied this effect for the CH_3OH isotopologue (S*19*, S*20*), while Siebert and Castillo-Chará (S*21*, S*22*) recently computed the torsion-vibration tunneling splittings. The band-splitting patterns in our Ne experiments of CH_3OH are similar to the observations by Perchard *et al.* For CH_3OD and CD_3OD, we do not observe splitting patterns that fit the torsion-vibration tunneling rationale.

In the following, we discuss the IR spectral assignment of the remaining spectral features that we attribute to methanol's pure vibration. The assignment uses the chemist and spectroscopist notation, as shown in **Table 1** and **Figure 4**. If not stated otherwise, the gas phase reference data is from Serralach et al. (S*12*)

Note that the notation for the torsion-vibration tunneling splitting comes from a model that describes the hydroxy-group as rotating in a periodic potential with a threefold barrier imposed by the methyl group. The basis functions for a quantum state within such a potential represent the C_3 point group. Thus, the torsion-vibration states are either in the *A* or *E* irreducible representation (S*15*, S*23*). On this score, a torsion-vibration tunneling transition has, e.g., the label *A→A*.

General Statements:

a) Perchard et al. reported **torsion-vibration tunneling splitting** for CH_3OH in Neon matrices by (S22). We observe this effect in our CH_3OH:Ne experiments.
b) Due to omnipresent water molecules, there is always a chance for **proton exchange in the methanol hydroxyl group,** leading to impurities. We generally observe pronounced CH_3OH impurities in the CH_3OD spectra and less pronounced CH_3OD impurities in the CH_3OH spectra. We generally observe pronounced CD_3OH impurities in the CD_3OD spectra. We did not study CD_3OH spectra.

c) Increasing methanol dilution in the host material reduces the chance of oligomerization. Still, **we observe oligomers**, probably mainly dimers, for the intense stretching vibrations.
d) All our spectra show no systematic pattern that molecular rotation could rationalize. Thus, we generally **rule out the rotation** of methanol in the matrix.
e) We can always assign one very pronounced band to a vibrational fundamental in all regions of the Argon spectra. The same holds for CH_3OD and CD_3OD species in Neon matrices, while splitting patterns for CH_3OH in Neon can be rationalized by (a). Thus, we generally **rule out matrix trapping sites** for methanol in both Argon and Neon matrices.

In the following, all numerical values are wavenumbers in cm^{-1}.

Region (I) - The *νCO* and *δOH* region (ν_6, ν_7, ν_8)

***νCO* symmetric stretch vibration o *A'* symmetry**	(ν_8 for CH_3OH, ν_7 for CH_3OD and CD_3OD)

- In the CH_3OH spectra, ν_8 is the most intense band (Ar: 1033.9, Ne: 1032.6). This band has weak sidebands at lower wavenumbers (Ar: 1027.7, Ne: 1027.3), likely due to oligomers and ^{13}C impurities.
- In the CH_3OH spectra, the weak band at slightly higher wavenumbers (Ar: 1043, Ne: 1040) is from CH_3OD impurities.
- In the CH_3OH spectra, some weak bands (Ar: 1054, Ne: 1051) remain unassigned. *
- In the CH_3OD spectra, ν_7 is the most intense band (Ar: 1042.8, Ne: 1039.9). This band has weak sidebands at lower wavenumbers (Ar: 1036.9, Ne: 1035.5), likely due to oligomers and ^{13}C impurities.
- In the CH_3OD spectra, the narrow band at slightly lower wavenumbers (Ar: 1034, Ne: 1033) is from CH_3OH impurities.
- In the CH_3OD spectra, some weak bands (Ar: 1056, Ne: 1052) remain unassigned. *
- In the CH_3OD spectra, some weak bands (Ar: 1045, Ne: 1042) remain unassigned. *
- In the CD_3OD spectra, ν_7 is *not* the most intense band (Ar: 983.0, Ne: 979.9). This band has weak sidebands at lower wavenumbers (Ar: 977.2, Ne: 974.1), likely due to oligomers and ^{13}C impurities.
- In the CD_3OD spectra, a relatively intense band at slightly higher wavenumbers (Ar: 988.4, Ne: 984.8) is from CD_3OH impurities. This band has weak sidebands at lower wavenumbers (Ar: 984, Ne: 981), likely due to oligomers and ^{13}C impurities.
- The observation of CD_3OH in the CD_3OD spectra is evidence for the proton exchange of the hydroxy group, probably with water.
- In the CD_3OD:Ne spectrum, some weak bands (991.1, 987.0) remain unassigned. *

** It is unlikely that these are matrix effects.*

***νCO* scaffold vibration of *A'* symmetry**	(ν_7 for CH_3OH, ν_6 for CH_3OD and CD_3OD)

- In the CH_3OH spectra, ν_7 is a low-intensity band (Ar: 1076.3, Ne: 1071.4).
- In the CH_3OH:Ne spectrum, this band has a splitting pattern. Perchard et al. (S22) show that this splitting pattern is due to torsion-vibration tunneling ($A \rightarrow A$: 1074.8, $E \rightarrow E$: 1071.4).
- In the CH_3OH:Ar spectrum, the splitting pattern (1076.3 and 1071.9) could be assigned similarly. However, we avoid this assignment because there is no evidence of torsion-vibration tunneling splitting in the residual Ar spectra.
- In the CH_3OD spectra, ν_6 is a low-intensity band (Ar: 1227.3, Ne: 1231.5). The intensity is so low that we can barely assign the band. Thus, we also have no evidence of tunneling splitting.
- For CD_3OD, we expect ν_6 from VCI calculations at 1021.8 cm^{-1} with very low intensity. It is somewhat problematic to find this band as our CD_3OD sample has slight impurities of CH_3OH and CH_3OD. The ν_6 band is likely to be overlapped by the *νCO* bands of CH_3OH and CH_3OD.

- In the CD_3OD:Ar spectrum, where the bands are generally broader, we cannot observe the ν_6 band. We thus rely on the value of 1031.5 published by Serralach et al.(S*12*).
- The bands are narrow in the CD3OD:Ne spectrum, and we observe ν_6 at 1028.7.

***δOH* in-plane bending vibration of *A'* symmetry** (ν_6 for CH_3OH, ν_8 for CH_3OD and CD_3OD)

- In the CH_3OH:Ar spectrum, ν_6 is a broad band at 1329.6.
- In the CH_3OH:Ne spectrum, torsion-vibration tunneling splitting (S*24*) results in two close bands with similar intensity (*A→A*: 1319.4, *E→E:* 1331.4) and a third at higher energy (*A→A(U₀)*: 1364.7).
- In the CH_3OD spectra, ν_8 is a strong band (Ar: 864.9, Ne: 864.1).
- In the CH_3OD:Ne spectra, a very weak band at 871.9 remains unassigned.
- In the CD_3OD spectra, ν_8 is a very strong band (Ar: 775.9, Ne: 774.4).
- In the CH_3OD:Ne spectra, a very weak band pair at 778.9/777.5 remains unassigned.

Region (II) - The δCH_3 region (ν_4, ν_5, ν_{10}, ν_{11})

$\delta_{as}CH_3$ "rocking" vibration of *A"* symmetry (ν_{11} for all three isotopologues)

This band is challenging to observe due to its low intensity.

- Lees et al. recently assigned the ν_{11} vibration at 1153.1 for CH_3OH in gas-phase spectroscopy (S*25*). We observe a very weak band for CH_3OH:Ar at 1162.8 and tentatively assign it as the ν_{11} vibration. For CH_3OH:Ne, we do not observe the ν_{11} vibration and rely on the assignment by at 1156.5 by Perchard et al. (S*19*).
- For CH_3OD in the gas phase, Falk et al. reported two δCH_3 vibrations: one of A'' symmetry to a weak band at 1228 and one of with A' symmetry to a very weak band at 1160 (S*26*). Later, Serralach et al. assigned the $\delta_{as}CH_3$ "rocking" vibration of *A"* symmetry in CH_3OD in gas-phase to 1142±4 and in Argon matrix to 1141.5. For both CH_3OD:Ar and CH_3OD:Ne, we do not observe the ν_{11} vibration. Thus, we rely on the literature data.
- For CD_3OD in the gas phase, Falk et al. reported a weak band at 892 without assignment and a band at 856 assigned to the $\delta_{as}CD_3$ "rocking" vibration of *A"* symmetry (S*26*).
- We tentatively reassign their observation. Their weak band at 892 is the $\delta_{as}CH_3$ vibration of CD_3OD because it matches our VCI calculation and MI-IR data. We observe a very weak band for CD_3OD:Ne at 890.3 but none for CD_3OD:Ar. For the latter, we take the observation of 895.0 by Serralach et al. (S*12*)

δ_sCH_3 "umbrella" vibration of *A'* symmetry (ν_5 for CH_3OH and CH_3OD, ν_4 for CD_3OD)

Although this band has relatively low intensity, we directly observe it in our Ne and Ar MI-IR spectra.

- For CD_3OD, we observe a shoulder (Ar: 1132.5, Ne: 1134.4) of a very intense band (Ar: 1127.9, Ne: 1129.0). According to Serralach et al., in Argon, the band at 1132.5 is from CD_3OD, while the band at 1127.9 is from the CD_3OH (S*12*). This assignment suggests a very high CD_3OH impurity in the CD_3OD sample. We know from the other regions of our CD_3OD spectrum that there are CD_3OH impurities. However, we are confident that CD_3OH is not in such an excess as the spectral pattern around 1130 would imply based on the assignment by Serralach et al.
- Hence, we reassign the ν_4 vibration of CD_3OD to the very intense band (Ar: 1127.9, Ne: 1129.0).
- For the other isotopologues, the assignment of ν_5 is more intricate.
- The CH_3OH:Ar spectra show a pattern of three well-separated broad bands with decreasing intensity (1451.8, 1450.2, 1447.5). The CH_3OD:Ar spectra show the same band (1451.7, 1450.0, 1447.1).
- The CH_3OH:Ne spectra show a broad band with two peaks (1450.5, 1448.1). The CH_3OD:Ne spectra show the same band shape (1450.3, 1448.1).

- For CH_3OH:Ne, the observed splitting is much larger (2.3 cm^{-1}) than the reported torsion-vibration tunneling splitting (0.3 cm^{-1}) by Perchard (S*19*). Also, for CH_3OH:Ar, we exclude torsion-vibration tunneling splitting, as there is no evidence for this effect in the complete CH_3OH:Ar spectrum.
- It is striking that hydroxyl deuteration neither affects the band shape nor shifts the wavenumbers in this spectral region, both in the Ar and Ne spectra. From the *vCO* and *vOH* regions, which comprise the most intense bands, we know that there are impurities of CH_3OH in the CH_3OD experiments and *vice versa*. Thus, the observed splitting patterns may be due to isotopic impurities.
- From VCI calculations, we estimate the ν_5 vibration to be roughly 1 cm^{-1} higher in CH_3OH than CH_3OD. Thus, we tentatively assign ν_5 of CH_3OH to the higher wavenumber (Ar: 1451.8, Ne: 1450.5) and ν_5 of CH_3OD to the lower wavenumber (Ar: 1450.0, Ne: 1448.1).

$\delta_s CH_3$ "scissor" vibration of *A'* symmetry (ν_4 for CH_3OH and CH_3OD, ν_5 for CD_3OD)
$\delta_{as} CH_3$ "wagging" vibration of *A"* symmetry (ν_{10} for all isotopologues)

- In the CH_3OH spectrum, ν_4 (Ar: 1473.5, Ne:1477.0) and ν_{10} (Ar: 1466.3, Ne:1469.0) are separated by about 8 cm^{-1}.
- In the CH_3OD spectrum, ν_4 (Ar: 1473.1, Ne:1473.4) and ν_{10} (Ar: 1466.2, Ne: 1476.6) are separated by about 6 cm^{-1}.
- In the CD_3OD spectrum, ν_5 (Ar: 1068.2, Ne: 1069.0) and ν_{10} (Ar: 1064.1, Ne: 1062.1) are separated by about 5 cm^{-1}.
- In the Ar spectra, hydroxyl deuteration neither affects the band shape nor shifts the wavenumbers, and we observe very similar bands for CH_3OH (ν_4: 1473.5, ν_{10}: 1466.3) and CH_3OD (ν_4: 1473.1, ν_{10}: 1466.2).
- For CH_3OH:Ne at a dilution of 1:250, we can dissect a broad band into a higher wavenumber domain with roughly four peaks (1478.4, 1477.0, 1474.4, and 1473.4) and a lower wavenumber domain with roughly three peaks (1469.0, 1468.1, and 1466.0 cm^{-1}). We assign the most intense peaks to ν_4 at 1477.0 cm^{-1} and ν_{10} at 1469.0 cm^{-1}. It is most likely that the other peaks are from torsion-vibration tunneling and oligomerization.
- For CH_3OH:Ne at a higher dilution of 1:1000, some of the bands in each domain disappear, most likely oligomer bands. The remaining bands (not marked in Figure 6) are very close to the torsion-tunneling splitting reported by Perchard et al. (S*19*). Here, we observe ν_4 (*A*→*E*: 1479.3, *E*→*A:* 1474.5) and ν_{10} (*A*→*E*: 1470.2, *E*→*A*: 1468.0).
- The CH_3OD:Ne spectrum shows very different behavior in this spectral region. The two domains show one peak for all dilutions, yielding a unique assignment of ν_4 (1473.4) and ν_{10} (1467.6). No splitting pattern emerges upon dilution, as in the case of CH3OH. Hence, torsion-vibration tunneling does not occur in CH_3OD.

Region (III) - The *vOH* and the "unambiguous" stretch region

***vOH* stretch vibration of *A'* symmetry** (ν_1 in CH_3OH and CD_3OD, ν_3 in CH_3OD)

The Argon MI-IR spectra are much simpler than the Neon MI-IR spectra and are straightforward to assign, analogous to Serralach et al. (S*12*). In the Neon MI-IR spectra, the CH_3OH shows a characteristic spectral pattern investigated previously regarding torsion-vibration splitting (S*19*) and oligomerization (S*27*). The Neon MI-IR spectra of CH_3OD and CD_3OD are similar in the *vOD* region. However, these spectra are less known, and we present first assignments here.

- In the CH_3OH:Ar spectrum, the ν_1 band peaks at 3666.7, with a split peak at 3669.0, and some weak oligomer/dimer bands can at around 3540 – 3520.
- In the CH_3OD:Ar spectrum, the ν_3 band peaks at 2704.8, with a split peak at 2706.6 and some weak oligomer/dimer bands at around 2605.

- In the CD_3OD:Ar spectrum, the ν_1 band peaks at 2706.8 and 2705.3, with the latter being more intense and taken for assignment. The band at 2605.6 is the ν_1 vibration of the dimer (S*6*).
- In the CH_3OH:Ne spectrum, we observe a multiply split band centered at 3690.1, with a splitting pattern due to torsion-vibration tunneling (*A*→*E*: 3694.9, *A*→*A*: 3690.1, *E*→*E*: 3688.0, *E*→*A*: 3686.1) (S*19*). Multiple oligomer/dimer bands are at around 3560 (S*27*). In experiments of low dilution (1:250), the oligomer bands are more intense than the monomer bands.
- In the CH_3OD:Ne spectrum, we observe a multiply split band (2721.7, 2719.6, 2718.2) peaking at 2721.7, which we assign to the ν_3 vibration of the monomer. There are further less intense bands (2716.5, 2714.2, 2713.2), which we have not assigned*****. The oligomer vibrations also occur as a multiply split band (2634.6, 2629.0, 2627.0, 2622.4). Those bands are most likely the νOD stretch of various oligomers of CD_3OD, probably also mixed with traces of CH_3OD and CH_3OH impurities (S*6*).
- In the CD_3OD:Ne spectrum, we observe a multiply split band (2723.0, 2720.5, 2719.0) peaking at 2723.0, which we assign to the ν_1 vibration of the monomer. There are further less intense bands (2716.8, 2714.8, 2713.7), which we have not assigned*****. The oligomer vibrations also occur as a multiply split band (2634.6, 2629.7, 2627.7, 2622.8).
- The evidence of oligomerization in this region further confirms that proton exchange can happen and explains the overall observation of non-deuterated impurities in our spectra of deuterated methanol.
  ***** At this point, it is not clear whether these are matrix effects or resonances.

$\nu_s CH_3$ symmetric stretch vibration of *A'* symmetry (ν_3 for CH_3OH and CD_3OD, ν_2 for CH_3OD)

- In the CH_3OH:Ar spectrum, ν_3 is a broad band peaking at 2848.0.
  In the CH_3OD:Ar spectrum, ν_2 is a broad band peaking at 2845.5.
- In the CH_3OH:Ne spectrum, ν_3 is a multiply split band peaking at 2841.5.
  In the CH_3OD:Ar spectrum, ν_2 is a multiply split band peaking at 2839.1.
- Hydroxyl deuteration redshifts the $\nu_s CH_3$ vibration by 3 cm^{-1} in Ar and 1.5 cm^{-1} in Ne spectra.
- In the CH_3OH:Ne spectrum, a pattern of three bands occurs (2848.6, 2841.5, 2839.2).
  We observe a similar pattern in the CH_3OD:Ne spectrum (2848.8, 2841.5, 2839.1).
- In the CH_3OD:Ne spectrum, the peak at 2839.1 is the most intense, and we assign it as ν_2 of CH_3OD. The occurrence of a similar peak in the CH_3OH:Ne spectrum is due to CH_3OD impurities.
- The remaining two peaks in the CH_3OH:Ne spectrum are due to torsion-vibration tunneling splitting of CH_3OH ν_3 (*A*→*E*: 2848.8, *E*→*A*: 2841.5) (S*19*).
- The same two peaks in the CH_3OD:Ne spectrum are due to torsion-vibration tunneling splitting of CH_3OH impurities.
- In the CH_3OH:Ne and the CH_3OD:Ne spectra, we each observe two further bands at lower wavenumbers (2831.0, 2825.6). Compared to each other, the band at 2831.0 is more intense in the CH_3OH:Ne spectrum, and the band at 2825.6 is more intense in the CH_3OD:Ne spectrum. As both bands vanish in high dilution, we assign them to oligomers/dimers.
- In the CD_3OD:Ar spectrum, ν_3 is a broad band peaking at 2078.0. The band shape is similar to the corresponding bands in CH_3OD:Ar and CH_3OH:Ar. We see no evidence for CD_3OH bands.
- In the CD_3OD:Ne spectrum, ν_3 is a multiply split band peaking at 2072.7. The band shape and the distance of 7 cm^{-1} between the peaks at 2079.7 and 2072.7 are very similar to what we observe for the torsion-vibration splitting in the CH_3OH:Ne spectrum, indicating that these peaks may arise from a similar effect. However, we do not expect torsion-vibration tunneling splitting for CD_3OD. We may assume the peaks at 2079.7 and 2072.7 to arise from CD_3OH impurities, as this species may very well show torsion-vibration splitting. Consequently, we attribute the 2072.7 peak to the ν_3 vibration of CD_3OD and CD_3OH.
- In the CD_3OD:Ne, we observe two bands at lower wavenumbers (2067.0, 2066.4). As both bands vanish in high dilution, we assign them to oligomers/dimers.

$\nu_s CH_3$ symmetric stretch vibration of *A′* symmetry (ν_2 for CH_3OH and CD_3OD, ν_1 for CH_3OD)

- In the CH_3OH:Ar spectrum, ν_2 is a broad band peaking at 3006.4.
- In the CH_3OD:Ar spectrum, ν_1 is a broad band peaking at 3006.9.
- Hydroxyl deuteration does not shift the wavenumbers, contrasting with the previously discussed $\nu_s CH_3$ around 2840.
- In the CH_3OH:Ne spectrum, ν_2 is a multiply split band centered at 2996.3. The splitting is due to torsion-vibration tunneling (*A*→*E*: 2996.3, *E*→*A*: 2993.8, *E*→*E*: 2991.3) (S*19*).
- In the CH_3OH:Ne spectrum, some peaks in this band remain unassigned (3007.3, 2986.5, 2982.3).
- In the CH_3OD:Ne spectrum, ν_1 is a broad band peaking at 2996.2. There is no sign of splitting due to torsion-vibration tunneling splitting, affirming the overall observation that there is no torsion-vibration tunneling in the case of a deuterated hydroxyl group.
- In the CH_3OD:Ne spectrum, some peaks in this band remain unassigned (3007.5, 2986.5, 2982.3).
- In the CD_3OD:Ar spectrum, ν_2 is a broad band peaking at 2255.0.
- In the CD_3OD:Ne spectrum, ν_2 is a broad band centered at 2247.3 with a pronounced sideband at 2240.9, probably due to oligomers.

Region (IV) - The "ambiguous" stretch region

We discuss this region in the main article (cf. Figure 3). In this section, we elaborate on technical aspects of the assignment, including the VCI resonance analysis. **Tables S3** to **S5** list the VCI analysis for the relevant spectral regions where resonances occur. Note that all VCI-calculated wavenumbers are w.r.t. the vibrational ground state and given in cm^{-1}.

$\nu_s CH_3$ symmetric stretch vibration of *A′* symmetry (ν_3 for CH_3OH and CD_3OD, ν_2 for CH_3OD)

- For CH_3OH (Ar: 2848.0, Ne: 2841.5), VCI computation (cf. **Table S3**) yields a state with a wavenumber of 2847.2 that has contributions from ν_3 (51.0%) and $2\nu_5$ (26.7%).
- For CH_3OD (Ar: 2845.5, Ne: 2839.1), VCI computation (cf. **Table S4**) yields a state with a wavenumber of 2844.2 that has contributions from ν_2 (57.2%) and $2\nu_5$ (27.4%).
- For CD_3OD (Ar: 2078.0, Ne: 2072.7), VCI computation (cf. **Table S5**) yields a state with a wavenumber of 2070.4 that has contributions from ν_3 (40.3%) and $2\nu_6$ (34.2%).
- The configurations associated with the fundamental (ν_2 or ν_3) always have the highest contribution, highlighting that our initial assignment is reasonable. However, to be precise, one should mention the resonance with another state where an overtone ($2\nu_5$ or $2\nu_6$) is also involved.

$\nu_{as} CH_2$ antisymmetric stretch vibration of *A″* symmetry (ν_9 for all isotopologues)

For this region, we encounter noteworthy ambiguities when relying on conventional notations.

For CH_3OH, ν_9 peaks at 2961.8 in Ar and 2966.9 in Ne, as historically established (S*12*). The VCI computation (cf. **Table S3**) yields a state with a wavenumber of 2960.8 with contributions from $\nu_4+\nu_{10}$ (59.9%) and ν_9 (28.3%). Consequently, labeling this state with $\nu_4+\nu_{10}$ rather than with ν_9 is reasonable. Alternatively, we acknowledge a resonance with a state where both $\nu_4+\nu_{10}$ and ν_9 are involved.

- The VCI computations (cf. **Table S3**) suggest three states probably involved in the resonance:
  - 2960.8 with contributions from $\nu_4+\nu_{10}$ (59.9%) and ν_9 (28.3%).
  - 2930.9 with contributions from $\nu_5+\nu_{10}$ (57.6%), $\nu_4+\nu_{10}$ (14.3%), and ν_9(19.7%).
  - 2914.0 with contributions from $\nu_5+\nu_{10}$ (35.0%), $\nu_4+\nu_{10}$ (16.2%), and ν_9 (33.0%).
- We can reasonably assign some bands to this resonance pattern in the experiment:
  - 2961.8 in Ar and 2966.9 in Ne,
  - 2930.0 in Ar and 2928.2 in Ne,
  - 2914.2 in Ar and 2912.0 in Ne.
- It remains impossible to uniquely assign those bands to one of the labels (ν_9, $\nu_5+\nu_{10}$, $\nu_4+\nu_{10}$).

For CH_3OD, ν_9 peaks at 2955.3 in Ar and 2953.6 in Ne. The VCI computation (cf. **Table S4**) yields a vibrational state with a wavenumber of 2910.4 that has a contribution from ν_9 (37.8%), $\nu_5+\nu_{10}$ (30.7%), and $\nu_4+\nu_{10}$ (18.0%). Consequently, it is reasonable to label this state as fundamental ν_9. However, the poor agreement with the experiment is not acceptable. Taking resonances into consideration clarifies the assignment.

- The VCI computations (cf. **Table S4**) suggest three states probably involved in the resonance:
  - 2959.5 with contributions from $\nu_4+\nu_{10}$ (64.1%) and ν_9 (26.7%).
  - 2930.1 with contributions from $\nu_5+\nu_{10}$ (63.9%), $\nu_4+\nu_{10}$ (11.1%), and ν_9 (17.5%).
  - 2910.4 with contributions from ν_9 (37.8%), $\nu_5+\nu_{10}$ (30.7%), and $\nu_4+\nu_{10}$ (18.0%)
- We can reasonably assign some bands to this resonance pattern in the experiment:
  - 2955.3 in Ar and 2953.6 in Ne,
  - 2920.9 in Ar and 2920.7 in Ne,
  - 2908.8 in Ar and 2905.5 in Ne.
- Again, it remains impossible to uniquely assign those bands to one of the labels (ν_9, $\nu_5+\nu_{10}$, $\nu_4+\nu_{10}$).

For CD_3OD, ν_9 peaks at 2218.9 in Ar and 2209.4 in Ne. The VCI computation (cf. **Table S5**) yields a vibrational state with a wavenumber of 2213.3 that has a contribution from ν_9 (64.4%) and from $\nu_5+\nu_{10}$ (12.6%). This result agrees with the experiment, and labeling this state as ν_9 is reasonable. However, resonance is still possible when looking at the VCI analysis in detail.

- The VCI computations (cf. **Table S5**) suggest three states probably involved in the resonance:
  - 2213.3 with contributions from ν_9 (64.4%) and $\nu_5+\nu_{10}$ (12.6%)
  - 2205.4 with contributions from $\nu_4+\nu_{10}$ (40.2%) and ν_9 (8.5%).
  - 2142.0 with contributions from $\nu_5+\nu_{10}$ (78.0%) and ν_9 (10.3%).
- We can reasonably assign two bands to this resonance pattern in the experiment:
  - 2218.9 in Ar and 2209.4 in Ne,
  - 2145 in Ar and 2144.3 in Ne.
- Again, it is not possible to uniquely assign those bands using the labels of the fundamental ν_9 and the two combination bands $\nu_5+\nu_{10}$, and $\nu_4+\nu_{10}$. However, there is a configuration with a leading contribution of 78%. Thus, the fully deuterated methanol is most straightforward to assign in terms of vibrational notations.

Overtones of	
***$\delta_s CH_3$* "umbrella" vibration of *A′* symmetry**	(2ν_5 for CH_3OH and CH_3OD, 2ν_4 for CD_3OD)
***$\delta_s CH_3$* "scissor" vibration of *A′* symmetry**	(2ν_4 for CH_3OH and CH_3OD, 2ν_5 for CD_3OD)
***$\delta_{as} CH_3$* "wagging" vibration of *A″* symmetry**	(2ν_{10} for all isotopologues)

In the "ambiguous" stretch region, there are three overtones of the bending vibrations that contribute to the resonance patterns and inhibit unique assignment, mainly for the CH_3OH and CH_3OD species.

Table S3: Resonances in the *νCH* region of the IR spectrum of $^{12}CH_3{}^{16}OH$

Experiment			VCI analysis												
Ar	Ne	Gas	State	Freq.	Int.	ν_2	ν_9	$2\nu_4$	$2\nu_{10}$	$2\nu_5$	$\nu_5+\nu_{10}$	$\nu_4+\nu_{10}$	$\nu_4+\nu_5$	$\nu_{12}+\nu_{11}+\nu_5$	ν_3
**2961.8**	**2966.9**	*2970±4*	ν_9	**2960.84**	29.87		28.3					59.9			
**2930.0**	**2928.2**	*2929.5*	ν_9	**2930.90**	17.64		19.7				57.6	14.3			
**2914.2**	**2912.0**	*2912±2*	ν_9	**2913.95**	28.01		33.0				35.0	16.2			
			ν_3	2959.80	14.46			50.4							12.8
			ν_3	2925.38	17.62					53.6					14.4
**2848.0**	**2841.5**	*2844.2*	ν_3	**2847.22**	27.06					26.7					51.0
			$2\nu_4$	2961.83	6.52			22.0						41.4	
**2955.9**	**2954.0**	*2956±4*	$2\nu_4$	**2955.07**	10.48			34.0						25.0	
			$2\nu_4$	2935.56	6.49			16.4	46.1				14.2		
			$2\nu_4$	2928.27	3.21			8.6	32.1	27.5			19.0		
**3006.4**	**2996.3**	*2999.0*	$2\nu_{10}$	3006.16	40.55	68.7			10.2						
			$2\nu_{10}$	2935.11	7.66			17.2	38.3				18.0		
n.o.	n.o.		$2\nu_{10}$	**2927.53**	3.23				40.4	26.6			14.4		
			$2\nu_5$	2927.33	2.27				37.4	27.2			16.0		
**2921.2**	**2918.9**	*2920±2*	$2\nu_5$	**2921.63**	14.14					22.6			50.7		
			$2\nu_5$	2847.04	26.69					25.5					50.4
2908.0	2905.6	*n.o.*													
n.o.	2895.7	*2893.2*													
			$\nu_4+\nu_{10}$	2960.59	30.43		28.8					58.9			
			$\nu_4+\nu_{10}$	2933.46	12.38		13.7				67.7	11.5			
			$\nu_4+\nu_{10}$	2916.01	32.12		37.8				25.5	19.5			
			$\nu_4+\nu_5$	2936.35	7.07			14.9	44.7				14.8		
			$\nu_4+\nu_5$	2928.23	4.61				25.6	32.2			13.0		
			$\nu_4+\nu_5$	2921.17	13.57					18.8			55.2		
			$\nu_5+\nu_{10}$	2930.81	17.83		20.0				56.9	14.7			
			$\nu_5+\nu_{10}$	2914.10	27.40		32.3				35.1	15.8			
			State	Freq.		ν_2	ν_9	$2\nu_4$	$2\nu_{10}$	$2\nu_5$	$\nu_5+\nu_{10}$	$\nu_4+\nu_{10}$	$\nu_4+\nu_5$	$\nu_{12}+\nu_{11}+\nu_5$	ν_3

Table S4: Resonances in the *vCH* region of the IR spectrum of $^{12}CH_3{}^{16}OD$

Experiment			VCI analysis													
Ar	Ne	Gas	State	Freq.	Int	ν_2	ν_9	$2\nu_4$	$2\nu_{10}$	$2\nu_5$	$\nu_5+\nu_{10}$	$\nu_4+\nu_{10}$	$\nu_4+\nu_5$	ν_1	ν_{11}	$\nu_{12}+\nu_8$
**2955.3**	**2953.6**	*2950±2*	ν_9	2959.52	28.27		26.7					64.1				
**2920.9**	**2920.7**	*2922.8*	ν_9	2930.13	15.85		17.5				63.9	11.1				
**2908.8**	**2905.5**	*2907.0*	ν_9	2910.42	32.07		37.8				30.7	18.0				
**2845.5**	**2839.1**	*2840.8*	ν_2	2844.18	30.38	57.2				27.4						
**2961.6**	**2966.5**	*2970±4*	ν_2	2960.16	16.68	14.6		59.2								
**2914.2**	**2913.8**		ν_2	2922.98	14.97	10.5				24.2			47.1			
			$2\nu_4$	2958.65	17.74	15.3		57.6		10.2						
**2927.9**	**2924.1**		$2\nu_4$	2933.34	6.82			20.3	42.2				12.9			
**2917.0**	**2917.6**		$2\nu_4$	2927.47	2.94			9.5	37.2	31.0			12.1			
	**2895.4**	*2896.5*														
			$2\nu_{10}$	2926.65	2.78				48.0	27.5						
			$2\nu_{10}$	2932.88	7.63			21.3	29.3	10.4			15.4			
			$2\nu_{10}$	3011.09	36.98				9.9					68.1		
			$2\nu_5$	2926.56	2.77				45.0	28.5						
			$2\nu_5$	2844.56	30.00	56.8				27.5						
			$2\nu_5$	2920.33	13.46					16.9			60.8			
			$2\nu_5$	2959.08	17.50	15.2		59.2		9.8						
			$2\nu_5$	2932.61	7.62			21.9	34.1	9.5			15.2			
			$\nu_4+\nu_{10}$	2959.34	28.77		27.2					63.6				
			$\nu_4+\nu_{10}$	2911.45	32.53		38.2				28.3	18.4				
			$\nu_4+\nu_{10}$	2931.12	14.37		15.8				66.5	10.5				
			$\nu_4+\nu_5$	2920.29	13.28					16.3			61.8			
			$\nu_4+\nu_5$	2933.47	7.73			19.3	41.5				13.0			
			$\nu_4+\nu_5$	2927.53	3.28				38.0	32.1			10.8			
			$\nu_5+\nu_{10}$	2929.95	16.11		17.8				63.2	11.3				
			$\nu_5+\nu_{10}$	2910.79	31.81		37.5				31.3	17.9				
			ν_{11}	1173.97	1.03										52.3	35.4
			ν_{11}	1137.16	1.29										42.1	38.4
			State	Freq.		ν_2	ν_9	$2\nu_4$	$2\nu_{10}$	$2\nu_5$	$\nu_5+\nu_{10}$	$\nu_4+\nu_{10}$	$\nu_4+\nu_5$		ν_{11}	$\nu_{12}+\nu_8$

Table S5: Resonances in the *νCD* region of the IR spectrum of $^{12}CD_3{}^{16}OD$

Experiment			VCI analysis																	
Ar	Ne	Gas	State	Freq.	Int.	ν_2	ν_9	$2\nu_6$	$2\nu_{10}$	$2\nu_5$	$\nu_5+\nu_{10}$	$\nu_4+\nu_{10}$	$\nu_7+\nu_6$	$\nu_5+\nu_4$	$\nu_6+\nu_4$	ν_3	$\nu_{12}+\nu_{11}+\nu_{10}$	$\nu_{12}+\nu_{11}+\nu_4$	$\nu_{12}+\nu_{11}+\nu_5$	$\nu_{12}+\nu_{11}+\nu_6$
**2255.0**	**2247.3**	*2250±2*	ν_2	2254.18	13.76	39.7												33.6		
			ν_2	2262.51		35.6												31.9		
**2218.9**	**2209.4**	*2212.6*	ν_9	2213.31	33.48		64.4				12.6									
**2145**	**2144.3**		ν_9	2141.98	3.24		10.3				78.0									
			ν_9	2205.44	4.40		8.5					40.2					38.0			
**2078.0**	**2072.7**	*2074.0*	ν_3	2070.00	16.91			33.5								40.7				
			ν_3	2087.00	16.66			37.6								32.8				
			ν_3	2158.44	4.45				10.3	69.8						9.5				
**2145**	**2147.7**		$2\nu_5$	2157.52	4.65				10.0	69.4										
**2132.5**	**2133.6**		$2\nu_5$	2140.22	1.36				74.7	8.8										
2132.5	2133.6		$2\nu_{10}$	2139.59	1.53															
			$2\nu_6$	2086.72	17.94			37.0								33.3				
			$2\nu_6$	2070.40	16.78			34.2								40.3				
			$2\nu_6$	2024.14	0.25			9.0					68.8							
n.o.	n.o.		$\nu_4+\nu_{10}$	2214.99	0.21							54.4					30.6			
n.o.	n.o.	*2208.5*	$\nu_4+\nu_{10}$	2204.85	3.23							36.6					41.8			
n.o.	n.o.		$\nu_4+\nu_5$	2218.63	0.04									51.1					30.8	
n.o.	n.o.		$\nu_4+\nu_5$	2209.44	0.06									37.5					44.9	
			$\nu_5+\nu_{10}$	2141.70	3.21															
n.o.	n.o.		$\nu_6+\nu_4$	2168.52	1.11										48.5					21.3
n.o.	n.o.		$\nu_6+\nu_4$	2182.41	0.22										20.6					43.1
			ν_4	1141.0	25.79															
			ν_4	1130.4	20.61															
			State	Freq.	Int.	ν_2	ν_9	$2\nu_6$	$2\nu_{10}$	$2\nu_5$	$\nu_5+\nu_{10}$	$\nu_4+\nu_{10}$	$\nu_7+\nu_6$	$\nu_5+\nu_4$	$\nu_6+\nu_4$	ν_3	$\nu_{12}+\nu_{11}+\nu_{10}$	$\nu_{12}+\nu_{11}+\nu_4$	$\nu_{12}+\nu_{11}+\nu_5$	$\nu_{12}+\nu_{11}+\nu_6$

Supplementary References

S1. K. Oenen, D. F. Dinu, K. R. Liedl, Determining internal coordinate sets for optimal representation of molecular vibration. *J. Chem. Phys.* **160** (2024), doi:10.1063/5.0180657.

S2. D. F. Dinu, M. Podewitz, H. Grothe, T. Loerting, K. R. Liedl, Decomposing anharmonicity and mode-coupling from matrix effects in the IR spectra of matrix-isolated carbon dioxide and methane. *Phys. Chem. Chem. Phys.* **22**, 17932–17947 (2020).

S3. E. Zak, J. Tennyson, O. L. Polyansky, L. Lodi, N. F. Zobov, S. A. Tashkun, V. I. Perevalov, A room temperature CO2 line list with ab initio computed intensities. *J. Quant. Spectrosc. Radiat. Transf.* **177**, 31–42 (2016).

S4. B. Stoicheff, High Resolution Raman Spectroscopy of Gases: XI. Spectra of CS2 and CO2. *Can. J. Phys.* **36**, 218–230 (1958).

S5. M. Van Thiel, E. D. Becker, G. C. Pimentel, Infrared Studies of Hydrogen Bonding of Methanol by the Matrix Isolation Technique. *J. Chem. Phys.* **27**, 95–99 (1957).

S6. A. J. Barnes, H. E. Hallam, Infra-red cryogenic studies. Part 4.—Isotopically substituted methanols in argon matrices. *Trans. Faraday Soc.* **66**, 1920–1931 (1970).

S7. L. Schriver, A. Burneau, J. P. Perchard, Infrared spectrum of the methanol dimer in matrices. Temperature and irradiation effects in solid nitrogen. *J. Chem. Phys.* **77**, 4926–4932 (1982).

S8. S. Coussan, N. Bakkas, A. Loutellier, J. P. Perchard, S. Racine, Infrared photoisomerization of the methanol cyclic trimer trapped in a nitrogen matrix. *Chem. Phys. Lett.* **217**, 123–130 (1994).

S9. S. Coussan, Y. Bouteiller, A. Loutellier, J. P. Perchard, S. Racine, A. Peremans, W. Q. Zheng, A. Tadjeddine, Infrared photoisomerization of the methanol dimer trapped in argon matrix: Monochromatic irradiation experiments and DFT calculations. *Chem. Phys.* **219**, 221–234 (1997).

S10. S. Coussan, A. Loutellier, J. P. Perchard, S. Racine, A. Peremans, A. Tadjeddine, W. Q. Zheng, Infrared laser induced isomerization of methanol polymers trapped in nitrogen matrix. I. Trimers. *J. Chem. Phys.* **107**, 6526–6540 (1997).

S11. S. Coussan, A. Loutellier, J. P. Perchard, S. Racine, A. Peremans, A. Tadjeddine, W. Q. Zheng, IR-induced interconversions between five conformers of methanol dimers trapped in nitrogen matrix. *Chem. Phys.* **223**, 279–292 (1997).

S12. A. Serrallach, R. Meyer, H. H. Günthard, Methanol and deuterated species: Infrared data, valence force field, rotamers, and conformation. *J. Mol. Spectrosc.* **52**, 94–129 (1974).

S13. J. S. Koehler, D. M. Dennison, Hindered Rotation in Methyl Alcohol. *Phys. Rev.* **57**, 1006–1021 (1940).

S14. J. O. Halford, Hindered rotation in methyl alcohol with note on ethyl alcohol. *J. Chem. Phys.* **18**, 361–365 (1950).

S15. R. M. Lees, J. G. Baker, Torsion-vibration-rotation interactions in methanol. I. Millimeter wave spectrum. *J. Chem. Phys.* **48**, 5299–5318 (1968).

S16. Y. Y. Kwan, D. M. Dennison, Analysis of the torsion-rotation spectra of the isotopic methanol molecules. *J. Mol. Spectrosc.* **43**, 291–319 (1972).

S17. L.-H. Xu, J. Fisher, R. M. Lees, H. Y. Shi, J. T. Hougen, J. C. Pearson, B. J. Drouin, G. A. Blake, R. Braakman, Torsion–rotation global analysis of the first three torsional states (vt=0, 1, 2) and terahertz database for methanol. *J. Mol. Spectrosc.* **251**, 305–313 (2008).

S18. Y.-P. Lee, Y.-J. Wu, R. M. Lees, L.-H. Xu, J. T. Hougen, Internal Rotation and Spin Conversion of CH 3 OH in Solid para-Hydrogen. *Science (80-.).* **311**, 365–368 (2006).

S19. J. P. Perchard, The torsion-vibration spectrum of methanol trapped in neon matrix. *Chem. Phys.* **332**, 86–94 (2007).

S20. J. P. Perchard, F. Romain, Y. Bouteiller, Determination of vibrational parameters of methanol from matrix-isolation infrared spectroscopy and ab initio calculations. Part 1 - Spectral analysis in the domain 11 000-200 cm-1. *Chem. Phys.* **343**, 35–46 (2008).

S21. J. Castillo-Chará, E. L. Sibert, Full dimensional theoretical study of the torsion-vibration eigenstates and torsional splittings of CH3OH. *J. Chem. Phys.* **119**, 11671–11681 (2003).

S22. E. L. Sibert, J. Castillo-Chará, Theoretical studies of the potential surface and vibrational spectroscopy of CH 3OH and its deuterated analogs. *J. Chem. Phys.* **122**, 0–11 (2005).

S23. C. C. Lin, J. D. Swalen, Internal rotation and microwave spectroscopy. *Rev. Mod. Phys.* **31**, 841–891 (1959).

S24. R. M. Lees, L. H. Xu, J. W. C. Johns, Z. F. Lu, B. P. Winnewisser, M. Lock, R. L. Sams, Fourier transform spectroscopy of CH3OH: Rotation-torsion-vibration structure for the CH3-rocking and OH-bending modes. *J. Mol. Spectrosc.* **228**, 528–543 (2004).

S25. R. M. Lees, L. H. Xu, Dark state illuminated: Infrared spectrum and inverted torsional structure of the v11 out-of-plane CH3-rocking mode of methanol. *Phys. Rev. Lett.* **84**, 3815–3818 (2000).

S26. M. Falk, E. Whalley, Infrared Spectra of Methanol and Deuterated Methanols in Gas, Liquid, and Solid Phases. *J. Chem. Phys.* **34**, 1554–1568 (1961).

S27. F. Kollipost, J. Andersen, D. W. Mahler, J. Heimdal, M. Heger, M. A. Suhm, R. Wugt Larsen, The effect of hydrogen bonding on torsional dynamics: A combined far-infrared jet and matrix isolation study of methanol dimer. *J. Chem. Phys.* **141** (2014), doi:10.1063/1.4900922.
